# ‘Can you recommend any good STI apps?’ A review of content, accuracy and comprehensiveness of current mobile medical applications for STIs and related genital infections

**DOI:** 10.1136/sextrans-2016-052690

**Published:** 2016-11-24

**Authors:** Jo Gibbs, Voula Gkatzidou, Laura Tickle, Sarah R Manning, Tilna Tilakkumar, Kate Hone, Richard E Ashcroft, Pam Sonnenberg, S Tariq Sadiq, Claudia S Estcourt

**Affiliations:** 1 Research Department of Infection and Population Health, University College London, London, UK; 2 Department of Design, Brunel University London, Uxbridge, UK; 3 Barts Sexual Health Centre, St Bartholomew's Hospital, Barts Health NHS Trust, London, UK; 4 Barts and the London School of Medicine and Dentistry, Queen Mary University of London, London, UK; 5 College of Engineering, Design and Physical Sciences, Brunel University London, Uxbridge, UK; 6 School of Law, Queen Mary University of London, London, UK; 7 Research Department of Infection & Population Health, University College London, London, UK; 8 Institute for Infection and Immunity, St George's, University of London, London, UK; 9 Blizard Institute, Barts and the London School of Medicine and Dentistry, Queen Mary University of London, London, UK

**Keywords:** SEXUAL HEALTH, INFORMATION TECHNOLOGY, COMMUNICATION TECHNOLOGIES

## Abstract

**Objective:**

Seeking sexual health information online is common, and provision of mobile medical applications (apps) for STIs is increasing. Young people, inherently at higher risk of STIs, are avid users of technology, and apps could be appealing sources of information. We undertook a comprehensive review of content and accuracy of apps for people seeking information about STIs.

**Methods:**

Search of Google Play and iTunes stores using general and specific search terms for apps regarding STIs and genital infections (except HIV), testing, diagnosis and management, 10 September 2014 to 16 September 2014. We assessed eligible apps against (1) 19 modified Health on The Net (HON) Foundation principles; and (2) comprehensiveness and accuracy of information on STIs/genital infections, and their diagnosis and management, compared with corresponding National Health Service STI information webpage content.

**Results:**

144/6642 apps were eligible. 57 were excluded after downloading. 87 were analysed. Only 29% of apps met ≥6 HON criteria. Content was highly variable: 34/87 (39%) covered one or two infections; 40 (46%) covered multiple STIs; 5 (6%) focused on accessing STI testing. 13 (15%) were fully, 46 (53%) mostly and 28 (32%) partially accurate. 25 (29%) contained ≥1 piece of potentially harmful information. Apps available on both iOS and Android were more accurate than single-platform apps. Only one app provided fully accurate and comprehensive information on chlamydia.

**Conclusions:**

Marked variation in content, quality and accuracy of available apps combined with the nearly one-third containing potentially harmful information risks undermining potential benefits of an e-Health approach to sexual health and well-being.

## Introduction

The popularity of mobile health applications (apps) is evident from the estimated 102 billion downloads of health-related apps worldwide.^1^ UK governmental health strategy strongly supports digitalisation of the National Health Service (NHS)^1^ with a focus on self-managed and remote care. One of the aims of the strategy is to develop a library of endorsed apps for a variety of medical conditions that would provide a user with reassurance on their quality and content. However, this is yet to be established.

The stigmatised nature of STIs and the technologically adept young adults they most affect (almost all young adults in the UK own an internet-enabled mobile phone (smartphone))^2^ might mean that provision of information about STIs, testing and treatment through apps is a highly effective and acceptable medium for those at highest risk. Evidence suggests that people often seek information about health-related issues online.^2^ In the UK, there is comprehensive, high-quality online information about many medical conditions in the form of an NHS-endorsed health website.^3^ However, in the absence of a robust framework specifically designed to evaluate mobile medical apps and current lack of any quality endorsements, the public have little guidance on accuracy and quality of the information they are accessing via apps. Erroneous or misleading clinical information could have consequences for both individual and public health.

Here we aimed to comprehensively evaluate the content and accuracy of currently available mobile medical native apps for STIs and related genital infections aimed at the general public by comparing app content with the information provided by NHS-endorsed websites (the gold standard for the purpose of this review).

## Methods

The methods we used were adapted from Huckvale *et al*,^4^ Abroms *et al*^5^ ^6^ and Muessig *et al*.^7^

### App search strategy

We searched Google Play and iTunes for free and paid apps on STIs and genital infections, testing, diagnosis and management between 10 September 2014 and 16 September 2014. Inclusion criteria included the following: the app addressed one or more aspects of sexual health promotion/safe sex advice, STI testing, diagnosis, management or support for partner notification; English language; available on UK Apple iOS or Google Play; free and paid apps; and multiplatform apps. Exclusion criteria included the following: specifically stated that it was not be regarded as a source of health-related information or apps categorised as ‘Entertainment’, ‘Games’, ‘Casual’ or ‘Puzzle’; apps that are specifically developed for healthcare professionals; absence of original content (ie, only links to secondary source); focused solely on HIV/AIDS, sexual positions, sexual performance, technique or sex trivia, sexual dysfunction, fertility and ovulation checker, contraception or condom size; general health or infection apps that do not specifically consider STIs or sexual health; apps that could not be downloaded because of country restrictions that prevented access in the UK; technical problems with the app after two attempts; sexual health clinic/condom locators outside the UK; paid apps that are a paid version of a free app (‘lite’ version); and app requires a username and password or creating an account to use it.

General search terms included the following: sexually transmitted diseases; STD; STDs; sexually transmitted infections; STI; STIs; sexual infection; sexual health; safe sex; safer sex; contraception. Specific search terms included the following: condoms; chlamydia; gonorrhoea; syphilis; human papillomavirus; HPV; genital warts; condylomata; herpes simplex virus; HSV; genital herpes; mycoplasma; *Mycoplasma genitalium*; non-specific urethritis; NSU; non-chlamydial non-gonococcal urethritis; NCNGU; pubic lice; crabs; trichomonas; *Trichomonas vaginalis*; shigella; shigellosis; pelvic inflammatory disease; epididymitis; balanitis.

### Assessment of apps

We developed a data extraction form^4^ (see web appendix tables 1–5) and downloaded eligible apps. Two researchers (JG and SRM or TT or LT) assessed each app independently on an Android mobile phone touch screen (Android apps) and on an iPhone 4S (iOS apps) and on both formats if applicable, according to the assessment criteria below. We piloted the data extraction form with five iOS apps and five Android apps and revised the form prior to conducting the full assessment. The researchers discussed any discrepancies in scoring, and a final score for each parameter agreed upon. Descriptive statistics were applied as appropriate using Microsoft Excel and Stata v13.

10.1136/sextrans-2016-052690.supp1 supplementary tables

Assessment criteria:

1. *General information*: Country of origin; number of downloads; rating; age restriction; theme; price; when app was last updated (see web appendix table 1).
2. *Compliance with modified Health on the Net (HON) Foundation principles*:^4^ ^8^ We used Huckvale's 18 quality standards for mobile medical apps^4^ that covered the following eight areas:
  - information provided authoritative (author named, training and qualification clearly stated);^4^
  - the purpose of the app (clearly stated that information is not a replacement for healthcare professional advice, app mission, purpose and audience stated, organisation behind app describe including its purpose and mission);
  - confidentiality (privacy policy stated);
  - information documented, referenced and dated (including medical content date of creation and modification present, grammar and spelling correct);
  - any claims are justified (all claims backed up with scientific evidence);
  - contact details, app operational and accessibility/presentation of information (method of contacting app publisher, app operational and information accessible and clearly stated);
  - funding (source of funding stated);
  - editorial and advertising policy.^4^

We added a further standard: Approval by NHS Choices Health App Library, a new online library containing apps that was launched as a pilot in 2013,^9^ as this postdated Huckvale's standards. We recorded the number and nature of the standards met by each app giving a possible maximum score of 19 (see web appendix table 2).

- *Assessment of focus, comprehensiveness and accuracy of apps:* Taking the perspective of a member of the public seeking information about STIs and related genital infections, we determined the degree to which each app provided accurate and comprehensive information about the relevant condition(s) by comparing the information the app contained with specifically designed patient and public information sources from three UK national health bodies: NHS Choices,^10^ ^11^ the British Association of Sexual Health and HIV 12 and the Family Planning Association (FPA)^13^ (see web appendix table 6). These were our ‘gold standard’ and contain comprehensive information on STI/genital infection management. At this stage, *Mycoplasma genitalium* was removed from the review as there was no information on this in any of these sources.
  - *Focus of each app*: We assessed the number of STIs and genital infections included in each app and the number of different aspects of diagnosis and management (see web appendix table 3), which were covered (parameters). This was then summarised to describe the proportion of apps that covered each infection and aspect of diagnosis and management.
  - *Comprehensiveness of parameters included in each app*: Each STI/genital infection and diagnosis and management parameter was then assessed for comprehensiveness of the content (see web appendix table 3). We classified comprehensiveness into three levels as compared with the gold standard: comprehensively covered (the app provided information on all or the majority (ie, >75% or three or more) aspects of the parameter); partially covered (the app covered one or more aspects of the parameter); not covered (provided no information).
  - *Accuracy of information included in each app*: We classified accuracy into four levels as compared with the gold standard (see web appendix table 4): *completely accurate* (all information is accurate); *majority accurate* (errors in only one aspect of the information (eg, testing) and no more than two minor errors); *partially accurate* (errors in more than one aspect of the information or more than two minor errors) and *not accurate* (completely inaccurate). In addition, if any app was found to contain information that we, as sexual health clinicians, felt could lead to physical harm or psychological distress to the user, we recorded this in the data extraction form.
- *Availability and nature of access to healthcare professionals*: Some apps permit engagement with healthcare professionals. Where appropriate, we recorded type of healthcare professional, methods of communication (email/phone/via app), ability to upload images and ability to share information with sex partner(s) (see web appendix table 5).

## Results

**Figure 1:**
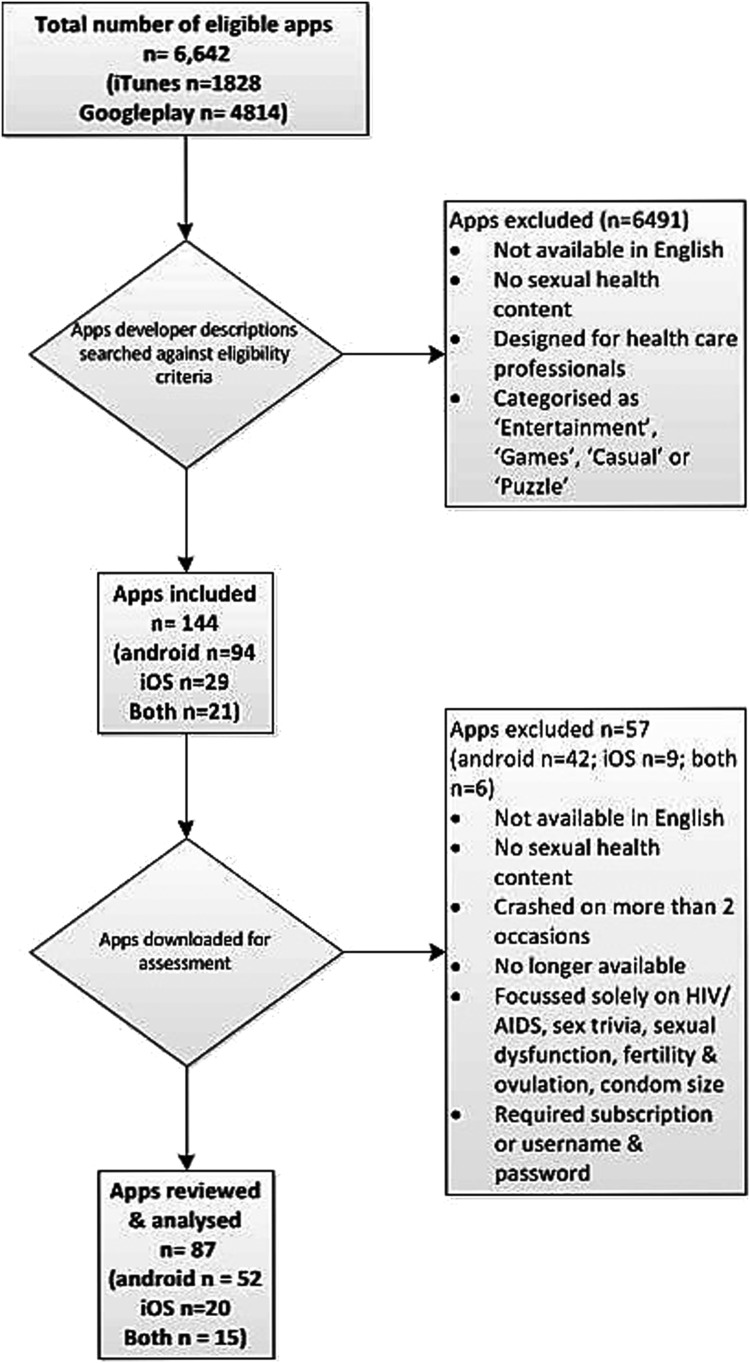
Flow chart.

We identified 6642 apps using the search terms, of which 144 were eligible, 57 were excluded after downloading and 87 were reviewed and analysed (figure 1).

### General information

UK was the most common country of origin (20%, 17/87 of apps), but 62% (54/87) of apps had no country of origin stated. Nearly half (48%, 25/52) of the apps were classified as ‘Health and Fitness’ and 21% (18/87) required users to pay a fee to access them. Approximately half of all apps had not been updated in the preceding 12 months, and therefore, potentially contained out-of-date information. The number of times each app had been downloaded was unavailable for iOS apps. Download of Android apps varied from 1 to >50 000 times (see web appendix tables 7–9).

### Modified HON criteria

**Figure 2:**
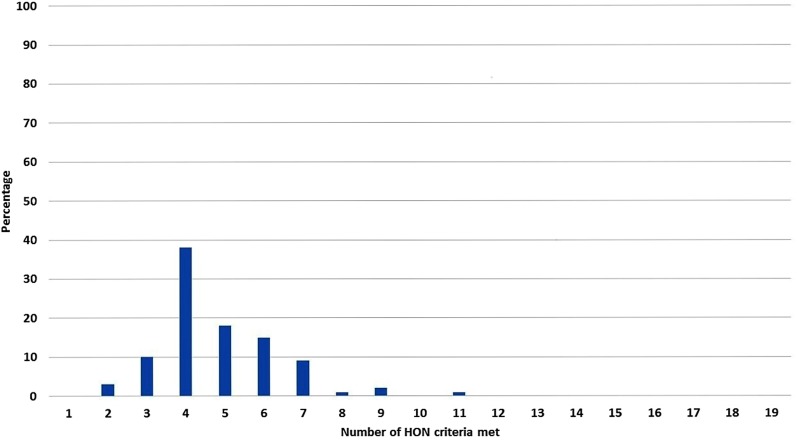
Proportion of Health on The Net (HON) criteria met.

The extent to which each app met each criterion is summarised in figure 2 and web appendix table 10. Overall, 71% (62/87) of apps met ≤5 of the 19 quality standards with the highest scoring app only meeting 11 of the 19 quality standards. No apps contained references to, or documentation of, where the information came from. Also, 2 of the 87 apps were in the NHS Choices Health Apps Library, and these scored 4 and 9.^10^

#### Focus and comprehensiveness of apps

**Figure 3:**
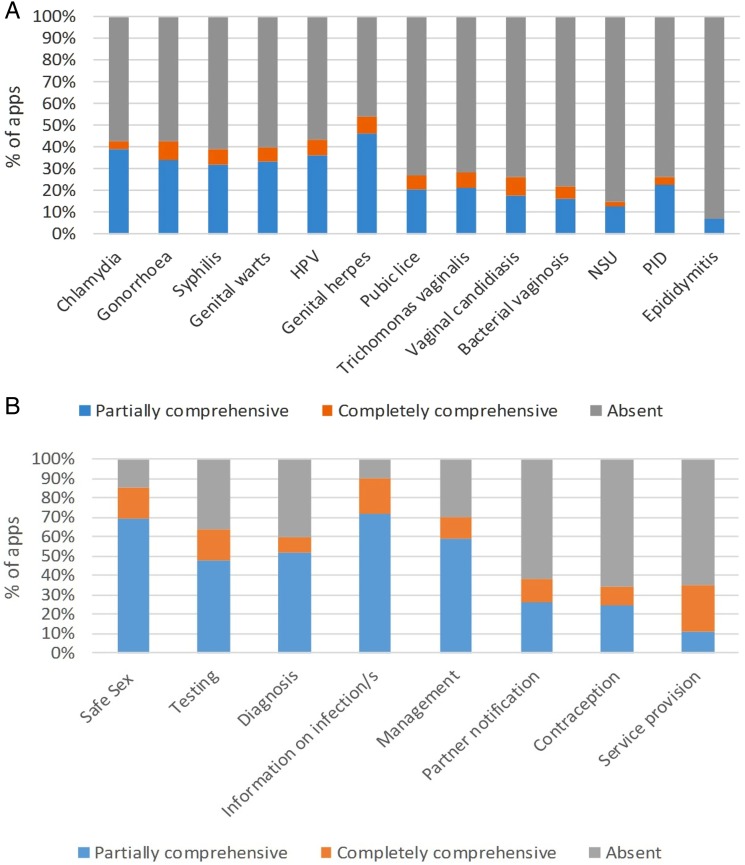
(A) Focus and comprehensiveness of different STIs and genital infections. (B) Focus and comprehensiveness of different aspects of STI/genital infection diagnosis and management. BV, bacterial vaginosis; HPV, human papillomavirus; NSU, non-specific urethritis; PID, pelvic inflammatory disease; PN, partner notification.

The focus of the apps was highly variable: 34/87 (39%) apps covered 1 or 2 infections, of these 16/34 (47%) were in the format of eBooks, predominately about genital herpes or candidiasis. Also, 40/87 (46%) covered multiple STIs and 5/87 (6%) apps focused solely on accessing STI testing (figure 3A).

Only a minority of apps provided fully comprehensive information on any individual parameter. Apps that were available in both platforms had broader focus and provided more comprehensive information compared with single-platform apps. There was great variability in focus and comprehensiveness of core elements of routine STI management (ie, safe sex, testing, diagnosis, information about STIs/genital infection, management, partner notification, contraception and service provision) in the apps reviewed (figure 3B and web appendix tables 11–14).

#### Accuracy of apps

**Figure 4:**
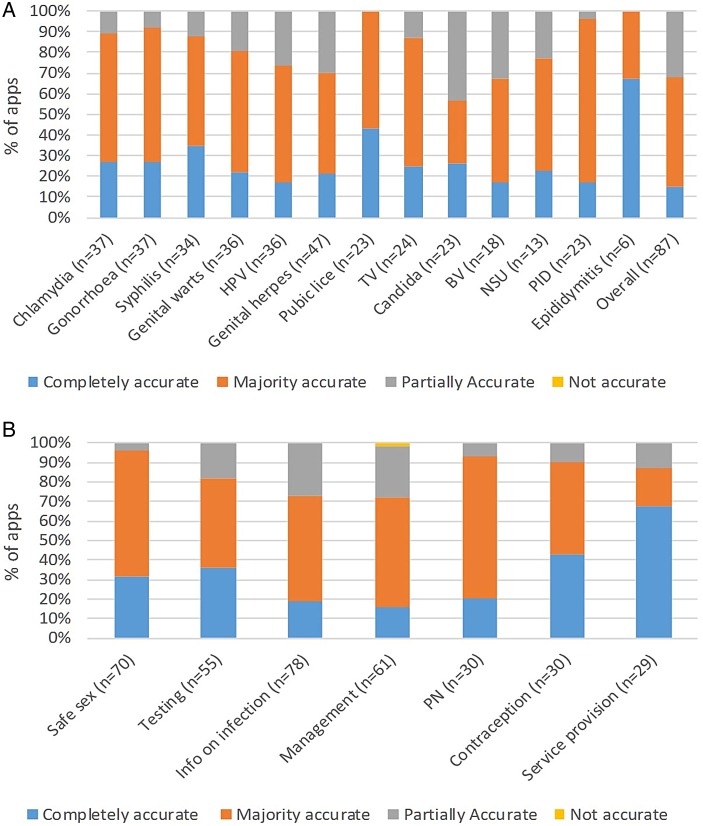
(A) Accuracy of information on individual STIs/genital infections and syndromes. (B) Accuracy of information on STI/genital diagnosis and management parameters. TV, *Trichomonas vaginalis.* BV, bacterial vaginosis; HPV, human papillomavirus; NSU, non-specific urethritis; PID, pelvic inflammatory disease; PN, partner notification.

There was wide variation in the accuracy of information provided in the apps (figure 4A, B and web appendix tables 15–18).

Overall, 13/87 (15%) apps were completely, 46/87 (53%) majority and 28/87 (32%) partially accurate. Apps available on both platforms, all of which had identical content between platforms, had a greater degree of accuracy than single-platform apps. Despite being the most commonly reported STI in the UK, only one app provided fully accurate and comprehensive information on chlamydia (FPA—find a clinic; see web appendices, App ID b3).

In total, 25/87 (29%) contained one or more instances of potentially harmful information. Indicative examples (web appendix tables 19–21) included the following:

Genital warts are bad. If they form in a bunch on your genitals, you will have a very bad time getting them treated and your relationships will shatter. (Web appendix table 20, App ID a15)

By sexual behaviour it does not only mean having vaginal intercourse. In fact, homosexuals can obtain this dreaded disease too through anal and oral sex. (Web appendix table 20, App ID a24)

Once women have left untreated with Chlamydia, they become highly likely of acquiring HIV or the human immunodeficiency virus. (Web appendix table 20, App ID a24)

Both the prescription drug Valtrex and some medicinal herbs have been proven to reduce herpes viral shedding in clinical studies … Certain medicinal herbs may also be beneficial in creating a strong immune response against HSV in non-infected partners. (Web appendix table 20, App ID a27)

Candida (found in yeast infections) can infect your blood, causing an overload of toxins to disrupt your system, wreaking havoc on your mind and body. (Web appendix table 20, App ID a51)

### Availability and nature of access to healthcare professionals

Only a small proportion of apps (13% (11/87)) allowed interaction with a healthcare professional (see web appendix tables 19–21), and the majority of these did not state the type of healthcare professional it would be (82% (9/11)). Only two apps allowed people to share information with their sexual partner through the app. No apps offered online clinical care such as diagnosis or management of STIs/genital infections, or the ability to access an electronic prescription to treat an STI via the app.

## Discussion

### Main findings

Although the initial search for apps about STIs and genital infections found thousands of hits, very few would meet the needs of a member of the public seeking accurate information about sexual health issues and their management—and these apps were difficult to identify. In contrast, when searching on the internet using a standard search engine, the first websites that come up are NHS endorsed. No apps documented where information came from, such that users had no way of assessing the reliability of the information provided. Most apps fell far short of recognised quality standards. This is particularly concerning when >40% of working-age adults are unable to fully understand and use health information.^14^

Although chlamydia, gonorrhoea, syphilis and genital warts were individually covered by approximately 40% of apps, only one app contained fully comprehensive, accurate information on chlamydia, UK's most common STI. Comprehensiveness of content was highly variable between apps and platforms. Most apps provided incomplete information on the parameters that they covered, and <20% provided fully comprehensive information on the STI/genital infections on which they focused. Similarly, accuracy of content was highly variable. Only a small proportion of apps contained completely accurate information, and almost one-third contained errors in more than one aspect of the information. The content of some apps was incorrect, condemnatory and scaremongering.

### Findings in relation to other literature

To our knowledge, this is the first review of apps focused on STIs and related conditions, which includes assessment of comprehensiveness and accuracy of content. The only other review in this field^7^ focused on characteristics and content of HIV and STI prevention and care apps but did not assess comprehensiveness and accuracy.

The inaccuracies within a high proportion of the apps we reviewed are of great concern. Currently, there is no easy way for the consumer to recognise apps that are likely to provide legitimate, trustworthy content. Although there has been some attempt to regulate mobile apps defined as ‘moderate-risk or high-risk medical devices’,^13^ ^15^ the vast majority of available mobile medical apps do not fall into these categories and therefore remain unregulated.^15^ ^16^ The difficulties in providing certification for app quality are well recognised, and some schemes have been suspended^17–19^ due to data security and feasibility issues.^20^ The components of the HON criteria that we used for this review are not necessarily applicable to all apps; for example, none of the apps we reviewed collected identifiable data, and therefore, one could question whether a privacy policy is required. Many now question the feasibility and usefulness of accreditation of medical and health apps^17–19^ ^21^ and fear that regulation will limit innovation through unnecessary bureaucracy, increased cost and delay in time to market.^19^ ^20^

Two apps included in the review were in the NHS Choices health apps library, and so should help patients in the absence of any other quality-accredited system. However, they only scored 4 and 9 when applying the adapted HON criteria. In addition, the reliability of the library has been recently called into question after finding ‘systematic gaps in compliance with data protection principles in accredited health apps’.^22^ The library is currently being upgraded and is not available.^23^

### Strengths and weaknesses

We conducted a wide-ranging, rigorous review using a framework that could be applied to many branches of medicine. However, the scope of the review, encompassing apps from the main mobile phone platforms and from a wide range of countries, meant that it was necessary to employ stringent inclusion and exclusion criteria. This included not extending the search to include HIV/AIDS. It is possible that we have missed relevant apps. In common with other app reviews, the speed of emergence of new apps means that it is difficult to conduct analyses and present findings before results become out of date.^19^ Despite rigorous assessment methods, it was not possible to completely eliminate the subjective element, particularly with respect to assessing the comprehensiveness and accuracy of app content. The need to assess a broad range of apps that focused on different aspects of sexual health and that had very different interfaces meant that any direct comparison between individual apps was impossible and that the parameters had to be kept broad. These difficulties have been highlighted previously.^24^

Ten apps required a subscription or a username and password; they either required one to have attended a clinic or asked for personal information. We were interested in generally accessible (open) apps rather than those designed for specific (closed) populations, and the researchers were using their own personal smartphones to access the apps, so we decided to exclude them. This could have biased findings through excluding either higher-quality or lower-quality apps.

### Recommendations and future work

Sexual health remains a stigmatised area, and people may find information provided in app format particularly appealing. However, many developers of content have not gone down the app route because users are not keen to download them on their phone. Instead, they have created content that uses responsive programming so that it can be accessed via Wi-Fi-enabled devices (eg, http://www.thedramadownunder.info/introduction). Although mobile apps have the potential to effect healthcare delivery,^25^ we are unlikely to see this potential reached unless we find a way of guiding users to assess the reliability of apps or directing them towards high-quality products. In the UK, NHS Choices app library could be improved by implementation of a robust, timely process for accreditation, clear quality standards and a process for maintaining and updating. However, this in itself presents challenges. Alternatively, we could focus on educating consumers on how to assess whether an app is a reliable source of information and recommending those apps that are known to be accurate and accessible.^17^ ^18^

**Key messages**

- STIs disproportionately affect young people, avid users of technology; the stigmatised nature of STIs means that accessing information using mobile apps maybe very appealing.
- It is difficult to identify good quality, relevant STIs and genital infections apps, and most apps reviewed fell short of recognised quality standards.
- Few apps meet the needs of people seeking comprehensive, accurate sexual health information. Content of a sizeable proportion of apps was incorrect and scaremongering.
- There is a pressing need for high-quality, easily identifiable apps that address the most common STI conditions and health concerns.
