# Supplementary material for: ‘Can you recommend any good STI apps?’ A review of content, accuracy and comprehensiveness of current mobile medical applications for STIs and related genital infections
Source: Sex Transm Infect. 2016 Nov 24;93(4):234–5. doi: 10.1136/sextrans-2016-052690 (PMC5520270; doi:10.1136/sextrans-2016-052690)
Supplement: supplementary tables [file sextrans-2016-052690supp001.pdf]

Web Table 1: Data extraction table for basic details

Section	Parameter	Coding	Description
Basic details	Title of App	Text	
	Developer	Text	Name of software publisher (e.g Googplay Apps)
	Country	Text	Country app designed in
	Region	Text	Region app designed for (e.g. Wirral)
	Version	Numerical	
	Number of downloads	Numerical	This is only available for Android apps
	Rating	Numerical	
	Number of ratings	numerical	
	Age restriction	text or numerical	Numerical for iOS, text for Android
	Theme (e.g. health and fitness)	Text	
	Price (£)	numerical (£0.00)	
	Last date updated	Date	
	Author's description of app	Text	What is stated in iTunes store or google play store.
	Our description of app	Text	This is your description of the app
	Target audience	1= general public 2 = people with STI 3= parents 4= other	
	Target audience_4	Text (99 =N/A)	
	Include/Exclude	1= include 2= exclude	
	Reason for exclusion	text (99=N/A)	
	General terms (e.g. sexual health)	text (99=absent)	This is a list of the general search terms that the app has been found under
	Specific terms (e.g. chlamydia)	text (99=absent)	This is a list of the specific search terms that the app has been found under
	Number of different terms apps appeared in	Numerical	This is the number of general terms and specific terms that the apps has been found under summed

Web Table 2: Data extraction of adapted HON criteria*

Section	Parameter	Coding	Description
Adapted HON Criteria	Author named	1 = yes 2= no	The actual author of the app named (not the publisher)
	Training stated	1 = yes 2= no	
	Qualification clearly stated	1 = yes 2 = no	
	Clearly stated that info is supportive and not a replacement	1 = completely 2= partially 3 = absent	
	App mission, purpose and audience stated	1 = completely 2= partially 3 = absent	
	Organisation behind app described, incl purpose and mission	1 = completely 2= partially 3 = absent	
	Privacy policy incl info on how emails are managed if used	1 = completely 2= partially 3 = absent	
	Documented, referenced and dated	1 = completely 2 = partially 3 = absent	
	Medical content date of creation present	1= yes 2 = no	
	Medical content date of modification present	1= yes 2= no	
	Grammar and spelling correct	1 = yes 2 = no	
	All claims backed up with scientific evidence	1 = completely 2= partially 3 = absent	
	App operational	1= yes 2= no	
	Information accessible and clearly stated	1= yes 2= no	
	Method of contacting app publisher	1 = yes 2= no	
	Source/s of funding stated	1 = yes 2 = no 3= not applicable	
	Conflicts of interest and external influences clearly stated in disclaimer	1 = yes 2 = no 3 = not applicable	
	Those with paying banners have advertising policy	1= yes 2= no 3= not applicable	
	Any conflict of interest explained	1 = yes 2 = no 3 = not applicable	
	Approved by NHS choices	1= yes 2=no	

*Health On the NET (HON) Foundation principles for health information on the internet

Web Table 3: Comprehensiveness of content

Section	Parameter	Coding	Description
Comprehensiveness of clinical information		1 = completely 2= partially 3 = absent	Completely = information on all or the majority (i.e. >75% or 3 or more) of aspects to do with parameter; Partial = information on 1 or more aspect to do with parameter but <75%; Absent = no information
	Safe sex	1 = completely 2= partially 3 = absent	Information on health promotion (e.g. condoms) and how to prevent onward transmission of STI/s
	Testing	1 = completely 2= partially 3 = absent	information on where and how you can get tested (blood test/ swabs at clinics etc.)
	Diagnosis	1 = completely 2= partially 3 = absent	what exactly the tests were/ how they were processed/ what they looked at
	Information about STIs/infection	1 = completely 2= partially 3 = absent	Information about 1 or more STI/infection including on aetiology/pathogenesis, symptoms, prevention, transmission, natural history
	Management	1 = completely 2= partially 3 = absent	Information about accessing treatment, what treatment is required, follow-up etc.
	Partner notification	1 = completely 2= partially 3 = absent	Information about the need to inform partners, abstaining from sex until partner treated, look back time for PN
	ePrescribing	1 = completely 2= partially 3 = absent	Able to get an ePrescription or information on this
	Contraception	1 = completely 2= partially 3 = absent	Information about different forms of contraception
	Service provision	1=completely 2=partially 3=absent	Information about where to access clinics, condoms, or contraceptive services.
	Other	Text	List any other aspects that are covered
	Chlamydia	1 = completely 2= partially 3 = absent	Completely = information on all or the majority (i.e. >75% or 3 or more) of aspects to do with parameter; Partial = information on 1 or more aspect to do with parameter but <75%; Absent = no information
	Gonorrhoea	1 = completely 2= partially 3 = absent	
	Mycoplasma	1 = completely 2= partially 3 = absent	
	Genital warts	1 = completely 2= partially 3 = absent	
	HPV	1 = completely 2= partially 3 = absent	
	Genital herpes	1 = completely 2= partially 3 = absent	
	Pubic lice	1=completely 2=partially 3=absent	
	Trichomonas vaginalis	1 = completely 2= partially 3 = absent	
	Vaginal candidiasis	1 = completely 2= partially 3 = absent	
	Bacterial vaginosis	1 = completely 2= partially 3 = absent	
	Non-specific urethritis	1 = completely 2= partially 3 = absent	
	Pelvic inflammatory disease	1 = completely 2= partially 3 = absent	
	Epididymitis	1=completely 2=partially 3=absent	

Web Table 4: Accuracy of clinical information

Section	Parameter	Coding	Description
Accuracy of clinical information	Safe sex	1= completely 2 = majority accurate 3 = partially accurate 4 = not accurate	Completely accurate = all information is accurate; Majority accurate = errors in only 1 aspect of the information (e.g. testing) or no more than 2 minor errors (i.e. will not impact on patient safety) throughout; Partially accurate = errors in more than 1 aspect of the information or more than 2 minor errors; Not accurate = completely inaccurate
	Testing	1= completely 2 = majority accurate 3 = partially accurate 4 = not accurate	
	Diagnosis	1= completely 2 = majority accurate 3 = partially accurate 4 = not accurate	
	Information about STIs/infection	1= completely 2 = majority accurate 3 = partially accurate 4 = not accurate	
	Management	1= completely 2 = majority accurate 3 = partially accurate 4 = not accurate	
	Partner notification	1= completely 2 = majority accurate 3 = partially accurate 4 = not accurate	
	ePrescribing	1= completely 2 = majority accurate 3 = partially accurate 4 = not accurate	
	Contraception	1= completely 2 = majority accurate 3 = partially accurate 4 = not accurate	
	Service provision	1= completely 2 = majority accurate 3 = partially accurate 4 = not accurate	Assess accuracy by checking at least 2 of clinics/sites listed are accurate
	Chlamydia	1= completely 2 = majority accurate 3 = partially accurate 4 = not accurate	Completely accurate = all information is accurate; Majority accurate = errors in only 1 aspect of the information (e.g. testing) or no more than 2 minor errors (i.e. will not impact on patient safety) throughout; Partially accurate = errors in more than 1 aspect of the information or more than 2 minor errors; Not accurate = completely inaccurate
	Gonorrhoea	1= completely 2 = majority accurate 3 = partially accurate 4 = not accurate	
	Syphilis	1= completely 2 = majority accurate 3 = partially accurate 4 = not accurate	
	Genital warts	1= completely 2 = majority accurate 3 = partially accurate 4 = not accurate	
	HPV	1= completely 2 = majority accurate 3 = partially accurate 4 = not accurate	
	Genital herpes	1= completely 2 = majority accurate 3 = partially accurate 4 = not accurate	
	Pubic lice	1= completely 2 = majority accurate 3 = partially accurate 4 = not accurate	
	Trichomonas vaginalis	1= completely 2 = majority accurate 3 = partially accurate 4 = not accurate	
	Vaginal candidiasis	1= completely 2 = majority accurate 3 = partially accurate 4 = not accurate	
	Bacterial vaginosis	1= completely 2 = majority accurate 3 = partially accurate 4 = not accurate	
	Non-specific urethritis	1= completely 2 = majority accurate 3 = partially accurate 4 = not accurate	
	Pelvic inflammatory disease	1= completely 2 = majority accurate 3 = partially accurate 4 = not accurate	
	Epididymitis	1= completely 2 = majority accurate 3 = partially accurate 4 = not accurate	
	Overall content accuracy	1= completely 2 = majority accurate 3 = partially accurate 4 = not accurate	

Web Table 5: Summary and additional information

Section	Parameter	Coding	Description
Additional information	App allows interaction with a healthcare professional	1 = yes 2 = no	
	Type of healthcare professional	1 = doctor 2 = nurse 3 = pharmacist 4 = other 99=N/A	Type of HCP app allows contact with
	Contact via email	1= yes 2 = no 99 = N/A	App allows contact via email
	Contact via phone	1= yes 2= no 99= N/A	App allows contact via phone
	Contact via app	1 = yes 2 = no 99= N/A	App allows contact via app
	Able to upload photo	1 = yes 2 = No 99 = N/A	
	Able to share info with SPs	1= yes 2= no	Able to share results/information on app with sexual partners
	Any other comments	Text	Any other comments that you think need mentioning that have not been covered

Web Table 6: Patient and public information sources

STI/Genital infection/ safe sex	BASHH patient information leaflet	NHS Choices webpage	Family planning association leaflet
Chlamydia		http://www.nhs.uk/conditions/Chlamydia/Pages/Introduction.aspx	http://www.fpa.org.uk/sites/default/files/chlamydia-information-and-advice.pdf
Gonorrhoea		http://www.nhs.uk/conditions/Gonorrhoea/Pages/Introduction.aspx	http://www.fpa.org.uk/sites/default/files/gonorrhoea-information-and-advice.pdf
Syphilis		http://www.nhs.uk/conditions/Syphilis/Pages/Introduction.aspx	http://www.fpa.org.uk/sites/default/files/syphilis-information-and-advice.pdf
Genital warts		http://www.nhs.uk/conditions/genital_warts/Pages/Introduction.aspx	http://www.fpa.org.uk/sites/default/files/genital-warts-information-and-advice.pdf
HPV		http://www.nhs.uk/conditions/vaccinations/pages/hpv-human-papillomavirus-vaccine.aspx http://www.nhs.uk/conditions/genital_warts/Pages/Introduction.aspx	http://www.fpa.org.uk/sites/default/files/genital-warts-information-and-advice.pdf
Genital herpes		http://www.nhs.uk/conditions/Genital-herpes/Pages/Introduction.aspx	http://www.fpa.org.uk/sites/default/files/genital-herpes-information-and-advice.pdf
Pubic lice		http://www.nhs.uk/conditions/pubic-lice/Pages/Introduction.aspx	http://www.fpa.org.uk/sites/default/files/pubic-lice-scabies-information-and-advice.pdf
Trichomonas vaginalis	http://www.bashh.org/documents/TV%20PIL%20Screen%20-%20Edit.pdf	http://www.nhs.uk/conditions/trichomonas_vaginalis/Pages/Introduction.aspx	http://www.fpa.org.uk/sites/default/files/trichomonas-vaginalis-information-and-advice.pdf

STI/Genital infection/ safe sex	BASHH patient information leaflet	NHS Choices webpage	Family planning association leaflet
Vaginal candidiasis		http://www.nhs.uk/conditions/thrush/pages/introduction.aspx	http://www.fpa.org.uk/sites/default/files/thrush-bacterial-vaginosis-information-and-advice.pdf
Bacterial vaginosis	http://www.bashh.org/documents/BV%20PIL%20Screen%20-%20Edit.pdf		http://www.fpa.org.uk/sites/default/files/thrush-bacterial-vaginosis-information-and-advice.pdf
Non-specific urethritis		http://www.nhs.uk/conditions/non_specific_urethritis/pages/causes.aspx	http://www.fpa.org.uk/sites/default/files/non-specific-urethritis-information-and-advice.pdf
Safe sex		http://www.nhs.uk/conditions/contraception-guide/pages/male-condoms.aspx http://www.nhs.uk/conditions/contraception-guide/pages/how-do-i-use-condom.aspx	http://www.fpa.org.uk/sites/default/files/oral-sex-and-sexually-transmitted-infections.pdf http://www.fpa.org.uk/sites/default/files/your-guide-to-contraception.pdf http://www.fpa.org.uk/sites/default/files/male-and-female-condoms-your-guide.pdf
General sexual health		http://www.nhs.uk/conditions/sexually-transmitted-infections/pages/introduction.aspx	http://www.fpa.org.uk/sites/default/files/your-sexual-health-where-to-get-help-and-advice.pdf
Pelvic inflammatory disease		http://www.nhs.uk/conditions/chlamydia/pages/complications.aspx http://www.nhs.uk/conditions/pelvic-inflammatory-disease/pages/introduction.aspx	
Epididymitis		http://www.nhs.uk/conditions/chlamydia/pages/complications.aspx	

Web Table 7: Summary of basic details (iOS only)

Platform	App ID	Title of App	Developer	Country	Number of downloads	Rating	Number of ratings	Age restriction	Theme (e.g. health & fitness)	Price (£)	Last date updated
iOS	i1	Sexually transmitted disease (STD) triage	iDoc24 AB	Sweden	99	No rating	0	12+	Medical	0	05/02/2014
iOS	i2	STD Guide	ViralMesh	USA	99	No rating	0	12+	Health & Fitness	0	17/11/2010
iOS	i3	STD Glossary	Space Monkeys LLC	USA	99	No rating	0	9+	Medical	0	02/06/2011
iOS	i4	iCondom Coventry	Raaza Ltd	UK	99	No rating	0	12+	Health & Fitness	0	11/07/2012
iOS	i5	99 - The Talk	Oneapp Application Studio Inc	USA	99	No rating	0	17+	Books	2.99	23/02/2010
iOS	i6	Safer Sex	Jo Langford	99	99	No rating	0	12+	Education	0	21/11/2013
iOS	i7	STD411	Frank Strona	America	99	No rating	0	17+	Health & Fitness	0	08/12/2010
iOS	i8	Private Girl Tips	KEYsci	USA	99	No rating	0	12+	Health & Fitness	1.49	26/03/2014
iOS	i9	SWISH APP	MyOxygen Limited	UK	99	No rating	0	12+	Medical	0	27/02/2013
iOS	i10	Sex Health Dictionary & Sexual Health Video Lessons	WindyApp Studio	99	99	No rating	0	17+	Health & Fitness	1.99	29/08/2014
iOS	i11	Safesex Guide	Mobile Identity Danmark Aps	Denmark	99	No rating	0	17+	Health & Fitness	1.49	16/02/2011
iOS	i12	Safe sex	ASD Soft	99	99	No rating	0	17+	Medical	0	19/06/2014
iOS	i13	SafeSex101	UCLA Student Media	USA	99	No rating	0	17+	Lifestyle	0	24/02/2013
iOS	i14	SAFE - Safety Awareness for Everyone	Harish Subrammaniam	99	99	No rating	0	17+	Health & Fitness	0	15/07/2014
iOS	i15	iSex - Sex Education and Terminology	Hassan Hosam	99	99	4	5	17+	Education	1.49	09/09/2009
iOS	i16	Girls's guide for sex myths	Soci solution	99	99	No rating	0	17+	Lifestyle	0.69	07/09/2011
iOS	i17	CaSH 2 U	ICE	UK	99	No rating	0	9+	Health & Fitness	0	17/07/2013
iOS	i18	Pap Test Lite	Elton Nallbati	99	99	No rating	0	4+	Medical	0	16/02/2013
iOS	i19	Natural Yeast Infection Solutions	minervaz	99	99	No rating	0	4+	Health & Fitness	2.49	10/02/2010
iOS	i20	A woman's guide to yeast infections	Aimfire LLC	99	99	No rating	0	12+	Health & Fitness	0.69	26/01/2010

Web Table 8: Summary of basic details (Android only)

Platform	App ID	Title of App	Developer	Country	Number of downloads	Rating	Number of ratings	Age restriction	Theme (e.g. health & fitness)	Price (£)	Last date updated
Android	a1	Abnormal Vaginal Discharge	App D Store	-	10	No rating	0	medium maturity	lifestyle	1.25	09/10/2011
Android	a2	About Herpes Simplex Infection	Nick Montano	-	50	No rating	0	low maturity	Health & Fitness	0	02/06/2014
Android	a3	After Sex	Sachem Software LLC	-	10,000	3.4	42	high maturity	Health & Fitness	0	19/08/2010
Android	a4	Bacterial Vaginosis Disease	Dmitry Grigorinov	-	10	No rating	0	everyone	medical	0	25/08/2014
Android	a5	Bacterial Vaginosis Guide	KoolAppz	-	50	5	1	everyone	Health & Fitness	3.07	05/10/2011
Android	a6	Bacterial Vaginosis Treatments	TokyInc	-	500	4.5	2	medium maturity	Health & Fitness	0	24/06/2014
Android	a7	Chlamydia Disease and Symptoms	Micheal Perterson	-	1	No rating	0	everyone	medical	0	24/08/2014
Android	a8	Chlamydia Know it Prevent it Treat it	Kindle Trove Apps	-	10	4	2	everyone	Health & Fitness	0	16/06/2014
Android	a9	Deadly Herpes Virus Acyclovir	WebHoldings	-	10	No rating	0	low maturity	Health & Fitness	0	21/06/2014
Android	a10	Female Herpes	IMJava Mobile	-	1000	4	3	medium maturity	medical	0	18/04/2013
Android	a11	Genital herpes guide	Ashi Company	-	10	4	1	high maturity	books and reference	0	28/07/2014
Android	a12	Genital Herpes Information	Naster Solomon	-	10	No rating	0	everyone	medical	0	20/08/2014
Android	a13	Genital Herpes Treatment	WebHoldings	-	100	4.7	3	medium maturity	Health & Fitness	0	13/06/2014
Android	a14	Genital Herpes Treatment	Ashi Company	-	10	No rating	0	high maturity	medical	0	29/07/2014
Android	a15	Genital Warts Guide	Havana Apps	-	100	No rating	0	low maturity	Health & Fitness	0	16/05/2014
Android	a16	Genital Warts Guide	Gooplay app	-	10	No rating	0	low maturity	Health & Fitness	0	05/06/2014
Android	a17	Genital warts info	Havana Apps	-	100	3	2	low maturity	books and reference	0	16/05/2014
Android	a18	Genital Warts Info	Gooplay app	-	100	No rating	0	low maturity	Health & Fitness	0	05/06/2014
Android	a19	Genital Warts Info	Ashi Company	-	10	2	1	medium maturity	books and reference	0	28/07/2014
Android	a20	Genital Warts Information	Naster Solomon	-	10-50	No rating	0	everyone	medical	0	20/08/2014
Android	a21	Get Rid of Bacterial Vaginosis	HealthSensei	-	500	5	4	low maturity	Health & Fitness	0	04/12/2013
Android	a22	Get Rid of Yeast Infection Now !	EclipseBoy	-	100	2	1	everyone	Health & Fitness	0	03/11/2013
Android	a23	Gonorrhea Disease & Symptoms	Naster Solomon	-	50	No rating	0	everyone	medical	0	20/08/2014
Android	a24	Guide to STDs	KoolAppz	-	10	1	1	everyone	Health & Fitness	3.11	13/07/2011
Android	a25	Herpes Knowledge	Gooplay app	-	50	No rating	0	low maturity	books and reference	0	15/05/2014
Android	a26	Herpes Lupus Psoriasis Eczema	Yoav Fael	-	100	4.5	4	everyone	Health & Fitness	0	01/06/2014
Android	a27	Herpes Treatment	KING APPS	-	500	4	4	everyone	Health & Fitness	0	30/05/2013

Web Table 8 continued

Platform	App ID	Title of App	Developer	Country	Number of downloads	Rating	Number of ratings	Age restriction	Theme (e.g. health & fitness)	Price (£)	Last date updated
Android	a28	HPV Infection Information	Naster Solomon	-	10	No rating	0	everyone	medical	0	26/08/2014
Android	a29	iGirl	pip90	Uganda	100	4.5	2	everyone	Health & Fitness	0	16/06/2013
Android	a30	Knowledge of Herpes	Gooplay app	-	10	No rating	0	low maturity	books and reference	0	05/06/2014
Android	a31	No Worries	Smartphone Media	UK	100	4.7	3	medium maturity	education	0	14/08/2013
Android	a32	NORISKS	dkberry	UK	50	No rating	0	everyone	Health & Fitness	0	22/07/2011
Android	a33	Painful urination in men	ConstantaSoft Inc	-	1	No rating	0	everyone	medical	4.33	05/04/2013
Android	a34	Pelvic inflammatory disease	thaweepong kongkratin	-	50	4	3	everyone	medical	0	23/08/2014
Android	a35	Protection - Sex	C-Dimensions Ltd	UK	5	No rating	0	medium maturity	lifestyle	0.59	24/02/2011
Android	a36	Pubic Lice Crabs Information	Noppawin sumongdee	-	10	4	1	everyone	medical	0	21/08/2014
Android	a37	SAFE	Amphibia	Malaysia	1000	4.3	34	medium maturity	Health & Fitness	0	20/05/2014
Android	a38	Safer sex	C-Dimensions Ltd	-	10	No rating	0	medium maturity	lifestyle	0.59	24/02/2011
Android	a39	Samedaydoctor - STD Testing	Creat Solutions (UK) Ltd	-	0	No rating	0	medium maturity	medical	0	20/08/2014
Android	a40	Sexual Education	Deep Powder Software	-	5	No rating	0	medium maturity	Health & Fitness	1.09	31/05/2010
Android	a41	Sexually transmitted Stds	noppawin sumongdee	-	100	2.5	4	everyone	medical	0	27/08/2014
Android	a42	Sheffield SH	Diva Creative	UK	100	No rating	0	low maturity	Health & Fitness	0	09/06/2014
Android	a43	STD glossary	Publish This, LLC	USA	10	4	1	everyone	medical	0.72	16/09/2013
Android	a44	Stop Vaginal Odor	Gallencraft	-	10000-50000	4	251	medium maturity	Health & Fitness	0	25/08/2014
Android	a45	Syphilis Disease and Symptoms	Pachara Kongsookdee	-	50	3	1	everyone	medical	0	22/08/2014
Android	a46	The Big Book - Symptoms of STD	Jak	-	5000	4.1	15	everyone	Health & Fitness	0	24/01/2013
Android	a47	The Sex Guide	C-Dimensions Ltd	-	10	No rating	0	high maturity	lifestyle	0.59	24/02/2011
Android	a48	Treat Genital Herpes Naturally	Muhhas	-	100	No rating	0	high maturity	Health & Fitness	0	06/04/2014
Android	a49	Trichomoniasis information	Pachara Kongsookdee	-	10	No rating	0	everyone	medical	0	25/08/2014
Android	a50	UCT Safe Sex	Information Systems Dept - University of Cape Town	-	5000-10000	4.2	53	medium maturity	Health & Fitness	0	09/05/2014
Android	a51	Yeast Infection	Dreamland Apps	-	50	No rating	0	everyone	books and reference	0	21/02/2014
Android	a52	Yeast Infection Home Remedy	Karl Evans	-	500	No rating	0	everyone	Health & Fitness	0	05/11/2012

Web Table 9: Summary of basic details available for both platforms

Platform	App ID	Title of App	Author	Country	Number of downloads	Android rating	iTune Rating	Android number of ratings	iTune Number of ratings	Android age restriction	iTune Age restriction	Android Theme	itune Theme (e.g. health & fitness)	Price (£)	iTune last date updated	Android Last date updated
Both	b1	C&SH Somerset	Scrumpylicious	UK	500-1000	4.7	No rating	7	8	Medium maturity	12+	Health & Fitness	Health& Fitness	0	16/08/2014	20/01/2015
Both	b2	Conifer Sex Health	Scrumpylicious	UK	100	4.2	No rating	4	99	Medium maturity	12+	health and fitness	health and fitness	0	14/01/2014	17/01/2014
Both	b3	FPA - Find a Clinic	FPA	UK	100	5	No rating	3	7	Medium maturity	12+	Medical	Medical	0	05/02/2014	05/02/2014
Both	b4	FREE 2 B ME	East Sussex County Council	UK	50 - 100	5	No rating	1	0	Medium maturity	12+	Health & Fitness	Health & fitness	0	22/02/2013	10/03/2013
Both	b5	Get Them Tested	Codigo Pte Ltd	Singapore	500 - 1,000	4	No rating	2	0	High maturity	12+	Health & Fitness	Health & fitness	0	12/05/2014	12/05/2014
Both	b6	Kent C Card	Kent Community Health NHS Trust	UK	500 - 1,000	3.7	No rating	3	10	Low maturity	4+	Health & fitness	Health & fitness	0	16/09/2013	16/09/2013
Both	b7	KIS-SK	CollegeMobile, Inc	Canada	100 - 500	4.7	No rating	3	0	Low maturity	4+	Medical	Medical	0	05/05/2014	05/05/2014
Both	b8	KYSH - Know Your Sexual Health	fixers	UK	10 to 50	No rating	No rating	0	0	High maturity	12+	Lifestyle	Health& Fitness	0	16/12/2013	22/11/2013
Both	b9	My Sex Doctor	MYSD LTD	UK	5000	3.7	No rating	73	0	High maturity	12+	Lifestyle	lifestyle	0	26/05/2014	26/05/2014
Both	b10	NeedTayKnow	99	UK	100	4.9	No rating	7	0	Medium maturity	12+	Medical	education	0	03/01/2014	03/01/2014
Both	b11	SexPositive	University of Oregon	USA	10,000 - 50,000	3.6	No rating	46	0	High maturity	17	Education	education	0	05/09/2014	04/09/2014
Both	b12	Sexual Health Guide	Global Internet Radio Technologies	Ireland	50,000 - 100,000	4	No rating	234	0	Medium maturity	17+	Education	education	0	24/07/2012	05/03/2013
Both	b13	Sexual Health Liverpool	Glow New Media Ltd	UK	50 - 100	4	No rating	1	0	Medium maturity	12+	Health& Fitness	Health& Fitness	0	09/04/2013	16/04/2013
Both	b14	Your rapid diagnosis STD	WWW Machealth Pty Ltd	Australia	50 - 100	0	0	0	0	Everyone	17	Medical	Medical	£2.99/£3.03	19/10/2011	29/01/2013
Both	b15	Your Choice Your Voice (YCYV)	Scrumpylicious	UK	50 - 100	4.5	0	2	0	Medium maturity	12+	health and fitness	health and fitness	0	07/10/2014	10/10/2014

Web Table 10: Summary of each HON criterion

		Author named (%)	Training stated (%)	Qualification clearly stated (%)	Clearly stated information supportive & not replacement (%)	App mission, purpose & audience stated (%)	Organisation behind app described, including purpose & mission (%)	Privacy policy including information on how emails are managed if used (%)	Documented, referenced & dated (%)	Medical content date of creation present (%)	Medical content date of modification present (%)	Grammar & spelling correct (%	All claims backed up with scientific evidence	App operational	Information accessible & clearly stated	Method of contacting app publisher	Source/s of funding stated	Conflicts of interest & externa influences clearly stated in disclaimer	Those with paying banners have advertising policy	Any conflict of interest explained	Approved by NHS choices
iOS n=20 (%)	Yes	8 (40)	3 (15)	2 (10)	5 (25)	10 (52.6)	6 (30)	2 (10)	0	0	0	18 (90)	0	19 (95)	17 (85)	8 (40)	2 (10)	0	0	0	0
	Partially	-	-	-	1 (5)	2 (10.5)	1 (5)	1 (5)	0	-	-	-	0	-	-	-	-	-	-	-	-
	No	12 (60)	17 (85)	18 (90)	14 (70)	7 (36.8)	13 (65)	17 (85)	20 (100)	20 (100)	20 (100)	2 (10)	20 (100)	1 (5)	3 (15)	12 (60)	18 (90)	19 (95)	3 (15)	1 (5)	20 (100)
	Not applicable	-	-	-	-	-	-	-	-	-	-	-	-	-	-	-	0	1 (5)	17 (85)	19 (15)	-
Android n=52 (%)	Yes	18 (35)	0	0	20 (38)	11 (21)	2 (4)	0	0	0	0	41 (79)	0	51 (98)	49 (94)	47 (90)	1 (2)	1 (2)	0	0	0
	Partially	-	-	-	4 (8)	26 (50)	6 (12)	0	2 (4)	-	-	-	2 (4)	-	-	-	-	-	-	-	-
	No	34 (65)	52 (100)	52 (100)	28 (54)	25 (29)	44 (85)	52 (100)	50 (96)	52 (100)	52 (100)	11 (21)	50 (96)	1 (2)	3 (6)	5 (10)	51 (98)	51 (98)	26 (50)	5 (10)	52 (100)
	Not applicable	-	-	-	-	-	-	-	-	-	-	-	-	-	-	-	0	0	26 (50)	47 (90)	-
Both n=15 (%)	Yes	0	0	0	4 (27)	12 (80)	8 (53)	1 (7)	0	0	0	14 (93)	0	15 (100)	11 (73)	9 (60)	1 (7)	0	1 (7)	0	2 (13)
	Partially	-	-	-	2 (13)	1 (7)	-	3 (20)	1 (7)	-	-	-	2 (13)	-	-	-	-	-	-	-	-
	No	15 (100)	15 (100)	15 (100)	9 (60)	2 (13)	7 (47)	11 (73)	14 (93)	52 (100)	52 (100)	1 (7)	13 (87)	0	4 (27)	6 (40)	14 (93)	12 (80)	0	0	13 (87)
	Not applicable	-	-	-	-	-	-	-	-	-	-	-	-	-	-	-	0	3 (20)	14 (93)	15 (100)	-
Total n=87 (%)	Yes	26 (30)	3 (3)	2 (2)	29 (33)	34 (39)	16 (18)	3 (3)	0	0	0	73 (84)	0	85 (97)	77 (89)	64 (74)	4 (5)	1 (1)	1 (1)	0	2 (2)
	Partially	-	-	-	7 (8)	29 (33)	14 (16)	4 (5)	3 (3)	-	-	-	4 (5)	-	-	-	-	-	-	-	-
	No	61 (70)	2 (84)	85 (98)	51 (59)	24 (28)	57 (66)	80 (92)	84 (97)	87 (100)	87 (100)	14 (16)	83 (95)	2 (2)	10 (11)	23 (26)	83 (95)	82 (94)	29 (33)	6 (7)	85 (98)
	Not applicable	-	-	-	-	-	-	-	-	-	-	-	-	-	-	-	0	4 (5)	57 (66)	81 (93)	-

Web Table 11: Comprehensiveness of different parameters

	Platform	Any coverage	Completely comprehensive	Partially comprehensive	Absent
Safe sex (%)	iOS n=20	16 (80)	1 (5)	15 (75)	4 (20)
	Android n=52	40 (77)	5 (10)	25 (48)	12 (23)
	Both n=15	13 (87)	7 (47)	6 (40)	2 (13)
	Total n=87	69 (79)	13 (15)	56 (64)	18 (21)
Testing (%)	iOS n=20	11 (55)	2 (10)	9 (45)	9 (45)
	Android n=52	32 (62)	5 (10)	27 (52)	20 (38)
	Both n=15	13 (87)	7 (47)	6 (40)	2 (13)
	Total n=87	56 (64)	14 (16)	42 (48)	31 (36)
Diagnosis (%)	iOS n=20	8 (40)	1 (5)	7 (35)	12 (60)
	Android n=52	34 (66)	4 (8)	30 (58)	18 (35)
	Both n=15	10 (66)	2 (13)	8 (53)	5 (33)
	Total n=87	52 (60)	7 (8)	45 (52)	35 (40)
Information about STIs/infection (%)	iOS n=20	15 (75)	3 (15)	12 (60)	5 (25)
	Android n=52	49 (94)	7 (13)	42 (81)	3 (6)
	Both n=15	14 (93)	6 (40)	8 (53)	1 (7)
	Total n=87	78 (89)	16 (18)	62 (71)	9 (10)
Management (%)	iOS n=20	10 (50)	1 (5)	9 (45)	10 (50)
	Android n=52	41 (79)	6 (12)	35 (67)	11 (21)
	Both n=15	10 (67)	3 (20)	7 (47)	5 (33)
	Total n=87	61 (70)	10 (11)	51 (59)	26 (30)
Partner notification (%)	iOS n=20	4 (20)	1 (5)	3 (15)	16 (80)
	Android n=52	18 (35)	5 (10)	13 (25)	34 (65)
	Both n=15	7 (47)	3 (20)	4 (27)	8 (53)
	Total n=87	29 (33)	9 (10)	20 (23)	58 (67)
ePrescribing (%)	iOS n=20	0	0	0	20 (100)
	Android n=52	0	0	0	52 (100)
	Both n=15	0	0	0	15 (100)
	Total n=87	0	0	0	87 (100)
Contraception (%)	iOS n=20	11 (55)	1 (5)	10 (50)	9 (45)
	Android n=52	9 (17)	1 (2)	8 (15)	43 (83)
	Both n=15	11 (73)	6 (40)	5 (33)	4 (27)
	Total n=87	30 (34)	8 (9)	22 (25)	57 (66)
Service provision (%)	iOS n=20	9 (45)	5 (25)	4 (20)	11 (55)
	Android n=52	9 (18)	3 (6)	6 (12)	43 (83)
	Both n=15	13 (87)	13 (87)	0	2 (13)
	Total n=87	31 (35)	21 (24)	10 (11)	56 (64)

Web Table 11 continued

STI/infection	Platform	Any coverage	Completely comprehensive	Partially comprehensive	Absent
Chlamydia (%)	iOS (n=20)	11 (55)	1 (5)	10 (50)	9 (45)
	Android (*n*=52)	14 (27)	0	14 (27)	38 (73)
	Both (n=15)	12 (80)	2 (13)	10 (67)	3 (20)
	Total (n=87)	37 (42)	3 (3)	34 (39)	50 (57)
Gonorrhoea (%)	iOS (n=20)	11 (55)	1 (5)	10 (50)	9 (45)
	Android (*n*=52)	14 (27)	1 (2)	13 (25)	38 (73)
	Both (n=15)	12 (80)	5 (33)	7 (47)	3 (27)
	Total (n=87)	37 (42)	7 (8)	30 (34)	50 (57)
Syphilis (%)	iOS (n=20)	11 (55)	1 (5)	10 (50)	9 (45)
	Android (*n*=52)	12 (23)	0	12 (23)	40 (77)
	Both (n=15)	11 (73)	5 (33)	6 (40)	4 (27)
	Total (n=87)	34 (39)	6 (7)	28 (32)	53 (61)
Genital warts (%)	iOS (n=20)	10 (50)	2 (10)	8 (40)	10 (50)
	Android (*n*=52)	14 (27)	0	14 (27)	38 (73)
	Both (n=15)	11 (73)	4 (27)	7 (47)	4 (27)
	Total (n=87)	35 (40)	6 (7)	29 (33)	52 (60)
HPV (%)	iOS (n=20)	11 (55)	2 (10)	9 (45)	9 (45)
	Android (*n*=52)	15 (29)	0	15 (29)	37 (71)
	Both (n=15)	11 (74)	4 (27)	7 (47)	4 (27)
	Total (n=87)	37 (43)	6 (7)	31 (36)	50 (57)
Genital herpes (%)	iOS (n=20)	11 (55)	1 (5)	10 (50)	9 (45)
	Android (*n*=52)	24 (46)	2 (4)	22 (42)	28 (54)
	Both (n=15)	12 (80)	4 (27)	8 (53)	3 (20)
	Total (n=87)	47 (54)	7 (8)	40 (46)	40 (46)
Pubic lice (%)	iOS (n=20)	7 (35)	0	7(35)	13 (65)
	Android (*n*=52)	8 (15)	1 (2)	7 (13)	44 (85)
	Both (n=15)	8 (53)	4 (27)	4 (27)	7 (47)
	Total (n=87)	23 (26)	5 (6)	18 (21)	64 (74)

STI/infection	Platform	Any coverage	Completely comprehensive	Partially comprehensive	Absent
Trichomonas vaginalis (%)	iOS (n=20)	7 (35)	1 (5)	6 (30)	13 (65)
	Android (*n*=52)	10 (19)	1 (2)	9 (17)	42 (81)
	Both (n=15)	7 (47)	4 (27)	3 (20)	8 (53)
	Total (n=87)	24 (28)	6 (7)	18 (21)	63 (72)
Vaginal candidiasis	iOS (n=20)	9 (45)	2 (10)	7 (35)	11 (55)
	Android (*n*=52)	8 (16)	2 (4)	6 (12)	44 (85)
	Both (n=15)	6 (40)	3 (20)	3 (20)	9 (60)
	Total (n=87)	23 (26)	7 (8)	16 (18)	64 (74)
Bacterial vaginosis	iOS (n=20)	2 (10)	0	2 (10)	18 (90)
	Android (*n*=52)	12 (23)	2 (4)	10 (19)	40 (77)
	Both (n=15)	5 (35)	3 (20)	2 (15)	10 (65)
	Total (n=87)	19 (22)	5 (6)	14 (16)	68 (78)
NSU	iOS (n=20)	3 (15)	0	3 (15)	17 (85)
	Android (*n*=52)	5 (10)	0	5 (10)	47 (90)
	Both (n=15)	5 (33)	2 (13)	3 (20)	10 (67)
	Total (n=87)	13 (15)	2 (2)	11 (13)	74 (85)
PID	iOS (n=20)	4 (20)	0	4 (20)	16 (80)
	Android (*n*=52)	15 (29)	1 (2)	14 (27)	37 (71)
	Both (n=15)	4 (27)	2 (13)	2 (13)	11 (73)
	Total (n=87)	23 (26)	3 (3)	20 (23)	64 (74)
Epididymitis	iOS (n=20)	3 (15)	0	3 (15)	17 (85)
	Android (*n*=52)	2 (4)	0	2 (4)	50 (96)
	Both (n=15)	1 (7)	0	1 (7)	14 (93)
	Total (n=87)	6 (7)	0	6 (7)	81 (93)

Web Table 12: Summary table of comprehensiveness of apps (iOS)

Platform	App ID	Title of App	Number of content parameters fully comprehensive	Number of STI/infections fully comprehensive	Number of content parameters partially comprehensive	Number of STI/infections partially comprehensive	No of content parameters covered total	Number of STI/infections covered total
iOS	i1	Sexually transmitted disease (STD) triage	2	0	5	7	7	7
iOS	i2	STD Guide	0	7	7	0	7	7
iOS	i3	STD Glossary	1	0	5	11	6	11
iOS	i4	iCondom Coventry	1	0	0	0	1	0
iOS	i5	99 - The Talk	3	2	3	11	6	13
iOS	i6	Safer Sex	0	0	2	0	2	0
iOS	i7	STD411	1	0	4	4	5	4
iOS	i8	Private Girl Tips	0	0	5	9	5	9
iOS	i9	SWISH APP	1	0	0	0	1	0
iOS	i10	Sex Health Dictionary & Sexual Health Video Lessons	0	0	6	12	6	12
iOS	i11	Safesex Guide	0	0	4	11	4	11
iOS	i12	Safe sex	0	0	2	0	2	0
iOS	i13	SafeSex101	2	0	5	9	7	9
iOS	i14	SAFE - Safety Awareness for Everyone	1	0	5	6	6	6
iOS	i15	iSex - Sex Education and Terminology	0	0	3	10	3	10
iOS	i16	Girls's guide for sex myths	1	0	2	0	3	0
iOS	i17	CaSH 2 U	1	0	1	0	2	0
iOS	i18	Pap Test Lite	0	0	4	1	4	1
iOS	i19	Natural Yeast Infection Solutions	0	1	3	0	3	1
iOS	i20	A woman's guide to yeast infections	1	1	3	0	4	1

Web Table 13: Summary table of comprehensiveness of apps (Android)

Platform	App ID	Title of App	Number of content parameters fully comprehensive	Number of STI/infections fully comprehensive	Number of content parameters partially comprehensive	Number of STI/infections partially comprehensive	No of content parameters covered total	Number of STI/infections covered total
Android	a1	Abnormal Vaginal Discharge	0	0	3	4	3	4
Android	a2	About Herpes Simplex Infection	0	0	3	1	3	1
Android	a3	After Sex	0	0	7	11	7	11
Android	a4	Bacterial Vaginosis Disease	0	1	5	1	5	2
Android	a5	Bacterial Vaginosis Guide	0	1	5	1	5	2
Android	a6	Bacterial Vaginosis Treatments	0	0	2	2	2	2
Android	a7	Chlamydia Disease and Symptoms	1	0	5	3	6	3
Android	a8	Chlamydia Know it Prevent it Treat it	0	0	2	2	2	2
Android	a9	Deadly Herpes Virus Acyclovir	0	0	4	1	4	1
Android	a10	Female Herpes	0	1	6	0	6	1
Android	a11	Genital herpes guide	0	0	3	1	3	1
Android	a12	Genital Herpes Information	0	0	6	1	6	1
Android	a13	Genital Herpes Treatment	0	0	5	1	5	1
Android	a14	Genital Herpes Treatment	0	0	5	1	5	1
Android	a15	Genital Warts Guide	1	0	3	2	4	2
Android	a16	Genital Warts Guide	0	0	3	2	3	2
Android	a17	Genital warts info	0	0	4	2	4	2
Android	a18	Genital Warts Info	2	0	4	2	6	2
Android	a19	Genital Warts Info	0	0	4	2	4	2
Android	a20	Genital Warts Information	1	0	4	2	5	2
Android	a21	Get Rid of Bacterial Vaginosis	1	0	1	1	2	1
Android	a22	Get Rid of Yeast Infection Now !	2	1	0	0	2	1
Android	a23	Gonorrhea Disease & Symptoms	1	1	5	2	6	3
Android	a24	Guide to STDs	0	0	5	10	5	10
Android	a25	Herpes Knowledge	1	0	1	1	2	1
Android	a26	Herpes Lupus Psoriasis Eczema	4	0	1	1	5	1

Web Table 13 continued

Platform	App ID	Title of App	Number of content parameters fully comprehensive	Number of STI/infections fully comprehensive	Number of content parameters partially comprehensive	Number of STI/infections partially comprehensive	No of content parameters covered total	Number of STI/infections covered total
Android	a27	Herpes Treatment	2	0	4	1	6	1
Android	a28	HPV Infection Information	0	0	5	2	5	2
Android	a29	iGirl	4	0	4	10	8	10
Android	a30	Knowledge of Herpes	0	0	2	1	2	1
Android	a31	No Worries	4	0	4	6	8	6
Android	a32	NORISKS	1	0	0	0	1	0
Android	a33	Painful urination in men	0	0	4	5	4	5
Android	a34	Pelvic inflammatory disease	0	1	7	2	7	3
Android	a35	Protection - Sex	1	0	6	6	7	6
Android	a36	Pubic Lice Crabs Information	1	1	5	0	6	1
Android	a37	SAFE	4	0	2	5	6	5
Android	a38	Safer sex	0	0	3	0	3	0
Android	a39	Samedaydoctor - STD Testing	2	0	1	9	3	9
Android	a40	Sexual Education	0	0	3	4	3	4
Android	a41	Sexually transmitted Stds	2	0	4	6	6	6
Android	a42	Sheffield SH	1	0	3	0	4	0
Android	a43	STD glossary	0	0	5	11	5	11
Android	a44	Stop Vaginal Odor	0	0	5	6	5	6
Android	a45	Syphilis Disease and Symptoms	0	0	6	1	6	1
Android	a46	The Big Book - Symptoms of STD	0	0	4	10	4	10
Android	a47	The Sex Guide	0	0	4	0	4	0
Android	a48	Treat Genital Herpes Naturally	0	1	5	0	5	1
Android	a49	Trichomoniasis information	0	1	6	0	6	1
Android	a50	UCT Safe Sex	0	0	2	0	2	0
Android	a51	Yeast Infection	0	0	1	1	1	1
Android	a52	Yeast Infection Home Remedy	0	1	4	0	4	1

Web Table 14: Summary table of comprehensiveness of apps (Both)

Platform	App ID	Title of App	Number of content parameters fully comprehensive	Number of STI/infections fully comprehensive	Number of content parameters partially comprehensive	Number of STI/infections partially comprehensive	No of content parameters covered total	Number of STI/infections covered total
Both	b1	C&SH Somerset	2	1	6	7	8	8
Both	b2	Conifer Sex Health	5	7	3	2	8	9
Both	b3	FPA - Find a Clinic	7	11	1	2	8	13
Both	b4	FREE 2 B ME	1	0	4	4	5	4
Both	b5	Get Them Tested	3	1	4	7	7	8
Both	b6	Kent C Card	1	0	3	0	4	0
Both	b7	KIS-SK	2	0	0	0	2	0
Both	b8	KYSH - Know Your Sexual Health	2	1	3	5	5	6
Both	b9	My Sex Doctor	5	10	2	2	7	12
Both	b10	NeedTayKnow	3	0	4	6	7	6
Both	b11	SexPositive	1	0	3	6	4	6
Both	b12	Sexual Health Guide	6	0	2	9	8	9
Both	b13	Sexual Health Liverpool	2	0	3	1	5	1
Both	b14	Your rapid diagnosis STD	6	11	1	0	7	11
Both	b15	Your Choice Your Voice (YCYV)	1	0	5	12	6	12

Web Table 15: Accuracy of different parameters

	Platform	Completely accurate	Majority accurate	Partially Accurate	Not accurate
Safe sex (%)	iOS (n=16)	6 (38)	8 (50)	2 (13)	0
	Android (*n* =41)	4 (10)	36 (88)	1 (2)	0
	Both (n=13)	13 (100)	0	0	0
	Total (n=70)	23 (31)	44 (63)	3 (4)	0
Testing (%)	iOS (n=12)	5 (42)	4 (33)	3 (25)	0
	Android (*n* =30)	3 (10)	20 (67)	7 (23)	0
	Both (n=13)	12 (92)	1 (8)	0	0
	Total (n=55)	20 (36)	25 (45)	10 (18)	0
Diagnosis (%)	iOS (n=8)	0	6(75)	2 (25)	0
	Android (*n* =34)	2 (6)	23 (68)	9 (27)	0
	Both (n=9)	7 (78)	1 (11)	1 (11)	0
	Total (n=51)	9 (18)	30 (59)	12 (24)	0
Information about STIs/infection (%)	iOS (n=15)	4 (27)	8 (53)	2 (20)	0
	Android (*n* =49)	3 (6)	29 (59)	17 (35)	0
	Both (n=14)	8 (57)	5 (36)	1 (7)	0
	Total (n=78)	15 (19)	42 (54)	21 (27)	0
Management (%)	iOS (n=10)	2 (20)	6 (60)	1 (10)	1 (10)
	Android (*n* =42)	0	27 (64)	15 (36)	0
	Both (n=9)	8 (89)	1 (11)	0	0
	Total (n=61)	10 (16)	34 (56)	16 (26)	1 (2)
Partner notification (%)	iOS (n=4)	1 (25)	3 (75)	0	0
	Android (*n* =19)	2 (11)	15 (79)	2 (11)	0
	Both	3 (43)	4 (57)	0	0
	Total (n=30)	6 (20)	22 (73)	2 (7)	0
ePrescribing (%)	iOS	0	0	0	0
	Android	0	0	0	0
	Both	0	0	0	0
	Total	0	0	0	0
Contraception (%)	iOS (n=11)	2 (18)	7 (64)	2 (18)	0
	Android (*n* =8)	1 (13)	6 (75)	1 (13)	0
	Both(n=11)	10 (91)	1 (9)	0	0
	Total (n=30)	13 (42)	14 (47)	3 (10)	0
Service provision (%)	iOS (n=9)	6 (67)	2 (22)	1 (11)	0
	Android (*n* =8)	4 (50)	3 (38)	1 (13)	0
	Both (n=12)	11 (92)	1 (8)	0	0
	Total (n=29)	21 (72)	6 (21)	4 (14)	0

Web Table 15 continued

STI/infection	Platform	Completely accurate	Majority accurate	Partially Accurate	Not accurate
**Chlamydia (%)**	iOS (n=11)	2 (18)	8 (73)	1 (9)	0
	Android (*n* =14)	0	12 (86)	2 (14)	0
	Both (n=12)	8 (67)	3 (25)	1 (8)	0
	Total (n=37)	10 (27)	23 (62)	4 (11)	0
**Gonorrhoea (%)**	iOS (n=11)	3 (27)	7 (64)	1 (9)	0
	Android (*n* =14)	0	12 (86)	2 (14)	0
	Both(n=12)	7 (58)	5 (41)	0	0
	Total (n=37)	10 (27)	24 (65)	3 (8)	0
**Syphilis (%)**	iOS (n=11)	3 (27)	6 (55)	2 (18)	0
	Android (*n* =12)	1 (8)	10 (83)	1 (8)	0
	Both (n=11)	8 (73)	2 (18)	2 (18)	0
	Total (n=34)	12 (35)	18 (53)	4 (12)	0
**Genital warts (%)**	iOS (n=10)	1 (10)	9 (90)	0	0
	Android (*n* =15)	0	8 (53)	7 (47)	0
	Both (n=11)	7 (64)	4 (36)	0	0
	Total (n=36)	8 (22)	21 (58)	7 (19)	0
**HPV (%)**	iOS (n=10)	2 (20)	7 (70)	1 (10)	0
	Android (*n* =15)	0	6 (40)	9 (60)	0
	Both	4 (36)	7 (64)	0	0
	Total (n=36)	6 (17)	20 (57)	10 (27)	0
**Genital herpes (%)**	iOS (n=11)	3 (27)	7 (64)	1 (9)	0
	Android (*n* =24)	0	12 (50)	12 (50)	0
	Both (n=12)	7 (58)	4 (33)	1 (8)	0
	Total (n=47)	10 (21)	23 (49)	14 (30)	0
**Pubic lice (%)**	iOS (n=7)	3 (43)	4 (57)	0	0
	Android (*n* =8)	0	8 (100)	0	0
	Both (n=8)	7 (88)	1 (13)	0	0
	Total	10 (43)	13 (57)	0	0

Web Table 15 continued

STI/infection	Platform	Completely accurate	Majority accurate	Partially Accurate	Not accurate
**Trichomonas vaginalis (%)**	iOS (n=7)	1 (14)	5 (71)	1 (14)	0
	Android (*n*=10)	0	8 (80)	2 (20)	0
	Both (n=7)	5 (71)	2 (29)	0	0
	Total (n=24)	6 (25)	15 (63)	3 (13)	0
**Vaginal candidiasis (%)**	iOS (n=9)	2 (22)	4 (44)	3 (33)	0
	Android (n=8)	1 (13)	2 (26)	5 (63)	0
	Both (n=6)	3 (50)	1 (17)	2 (33)	0
	Total (n=23)	6 (26)	7 (30)	10 (43)	0
**Bacterial vaginosis (%)**	iOS (n=2)	1 (50)	1 (50)	0	0
	Android (*n*=11)	0	6 (55)	5 (45)	0
	Both (n=5)	2 (40)	2 (40)	1 (20)	0
	Total (n=18)	3 (17)	9 (50)	6 (33)	0
**NSU (%)**	iOS (n=3)	1 (33)	2 (67)	0	0
	Android (*n*=5)	0	4 (80)	1 (20)	0
	Both (n=5)	2 (40)	1 (20)	2 (40)	0
	Total	3 (23)	7 (54)	6 (46)	0
**PID (%)**	iOS (n=4)	1 (25)	3 (75)	0	0
	Android (*n*=15)	1 (7)	13 (87)	1 (7)	0
	Both (n=4)	2 (50)	2 (50)	0	0
	Total (n=23)	4 (17)	18 (78)	1 (4)	0
**Epididymitis (%)**	iOS (n=20)	2 (67)	1 (33)	0	0
	Android (*n*=2)	1 (50)	1(50)	0	0
	Both (n=1)	1 (100)	0	0	0
	Total (n=6)	4 (67)	2 (33)	0	0
**Overall (%)**	iOS (n=20)	6 (30)	11 (55)	3 (15)	0
	Android (*n*=52)	0	28 (54)	24 (46)	0
	Both (n=15)	7 (47)	7 (47)	1 (7)	0
	Total (n=87)	13 (15)	46 (53)	28 (32)	0

Web Table 16: Summary table of accuracy of apps (iOS)

Platform	App ID	Title of App	Sum of content type accuracy scores	Content type accuracy %	Sum of STI/infection accuracy scores	STI/infection accuracy %	Overall Accuracy score	Overall Accuracy %
iOS	i1	Sexually transmitted disease (STD) triage	13	97	15	97	28	97
iOS	i2	STD Guide	37	45	39	40	76	43
iOS	i3	STD Glossary	28	77	36	83	64	80
iOS	i4	iCondom Coventry	2	100	-	-	-	-
iOS	i5	99 - The Talk	13	97	27	99	40	98
iOS	i6	Safer Sex	8	67	-	-	-	-
iOS	i7	STD411	10	100	8	100	18	100
iOS	i8	Private Girl Tips	17	76	29	80	46	
iOS	i9	SWISH APP	2	100	-	-	-	
iOS	i10	Sex Health Dictionary & Sexual Health Video Lessons	17	87	35	80	52	83
iOS	i11	Safesex Guide	16	67	46	69	62	68
iOS	i12	Safe sex	8	67	-	-	-	-
iOS	i13	SafeSex101	20	85	32	75	52	80
iOS	i14	SAFE - Safety Awareness for Everyone	24	76	21	75	45	75
iOS	i15	iSex - Sex Education and Terminology	12	67	31	81	43	74
iOS	i16	Girls's guide for sex myths	10	77	-	-	-	-
iOS	i17	CaSH 2 U	4	100	-	-	-	-
iOS	i18	Pap Test Lite	16	67	4	67	20	67
iOS	i19	Natural Yeast Infection Solutions	16	44	6	37	22	39
iOS	i20	A woman's guide to yeast infections	22	60	6	37	28	47

Web Table 17: Summary table of accuracy of apps (Android)

Platform	App ID	Title of App	Sum of content type accuracy scores	Content type accuracy %	Sum of STI/infection accuracy scores	STI/infection accuracy %	Overall Accuracy score	Overall Accuracy %
Android	a1	Abnormal Vaginal Discharge	10	78	18	63	28	67
Android	a2	About Herpes Simplex Infection	12	67	6	33	18	58
Android	a3	After Sex	23	79	35	80	58	80
Android	a4	Bacterial Vaginosis Disease	15	83	6	83	21	83
Android	a5	Bacterial Vaginosis Guide	26	47	8	67	34	52
Android	a6	Bacterial Vaginosis Treatments	10	50	9	58	19	54
Android	a7	Chlamydia Disease and Symptoms	17	86	9	83	26	85
Android	a8	Chlamydia Know it Prevent it Treat it	16	67	6	83	22	72
Android	a9	Deadly Herpes Virus Acyclovir	16	67	6	33	22	60
Android	a10	Female Herpes	24	67	3	83	27	69
Android	a11	Genital herpes guide	14	56	5	50	19	54
Android	a12	Genital Herpes Information	19	81	3	83	22	81
Android	a13	Genital Herpes Treatment	24	53	5	50	29	53
Android	a14	Genital Herpes Treatment	25	50	5	50	30	50
Android	a15	Genital Warts Guide	13	61	11	43	24	53
Android	a16	Genital Warts Guide	12	67	10	50	22	60
Android	a17	Genital warts info	23	38	12	33	35	36
Android	a18	Genital Warts Info	22	47	12	33	34	39
Android	a19	Genital Warts Info	23	38	12	33	35	36
Android	a20	Genital Warts Information	18	83	6	83	24	83
Android	a21	Get Rid of Bacterial Vaginosis	12	33	6	33	18	33
Android	a22	Get Rid of Yeast Infection Now !	12	33	6	33	18	33
Android	a23	Gonorrhea Disease & Symptoms	17	86	7	95	24	89
Android	a24	Guide to STDs	23	57	44	64	67	64
Android	a25	Herpes Knowledge	10	50	5	50	15	50
Android	a26	Herpes Lupus Psoriasis Eczema	11	97	3	83	14	94

Web Table 17 continued

Platform	App ID	Title of App	Sum of content type accuracy scores	Content type accuracy %	Sum of STI/infection accuracy scores	STI/infection accuracy %	Overall Accuracy score	Overall Accuracy %
Android	a27	Herpes Treatment	24	66.7	5	50	29	64
Android	a28	HPV Infection Information	15	83.3	6	83.3	21	83
Android	a29	iGirl	21	83.3	34	76.7	55	80
Android	a30	Knowledge of Herpes	11	41.7	5	50	16	44
Android	a31	No Worries	23	85.4	18	83.3	41	85
Android	a32	NORISKS	2	100	-	-	-	-
Android	a33	Painful urination in men	16	80	20	66.7	36	73
Android	a34	Pelvic inflammatory disease	21	83.3	9	83.3	30	83
Android	a35	Protection - Sex	17	92.9	21	75	38	85
Android	a36	Pubic Lice Crabs Information	16	66.7	3	83.3	19	81
Android	a37	SAFE	22	72.2	21	75.1	43	74
Android	a38	Safer sex	8	88.9	-	-	-	-
Android	a39	Samedaydoctor - STD Testing	11	72.2	26	79.1	37	80
Android	a40	Sexual Education	14	55.6	15	62.5	29	64
Android	a41	Sexually transmitted Stds	21	74.6	21	75	42	75
Android	a42	Sheffield SH	9	83.3	-	-	-	-
Android	a43	STD glossary	16	80	42	69.7	58	73
Android	a44	Stop Vaginal Odor	28	40	35	72	63	38
Android	a45	Syphilis Disease and Symptoms	18	83.3	3	83.3	21	83
Android	a46	The Big Book - Symptoms of STD	12	83.3	31	81.7	43	82
Android	a47	The Sex Guide	18	73.3	-	-	-	-
Android	a48	Treat Genital Herpes Naturally	19	63.3	5	50	24	67
Android	a49	Trichomoniasis information	18	83.3	3	83.3	21	83
Android	a50	UCT Safe Sex	6	83.3	-	-	-	-
Android	a51	Yeast Infection	5	50	5	50	10	50
Android	a52	Yeast Infection Home Remedy	19	54.2	6	33.3	25	50

Web Table 18: Summary table of accuracy of apps (Both)

Platform	App ID	Title of App	Sum of content type accuracy scores	Content type accuracy %	Sum of STI/infection accuracy scores	STI/infection accuracy %	Overall Accuracy score	Overall Accuracy %
Both	b1	C&SH Somerset	19	94	18	96	37	95
Both	b2	Conifer Sex Health	18	96	19	98	37	97
Both	b3	FPA - Find a Clinic	16	100	26	100	42	100
Both	b4	FREE 2 B ME	13	90	16	89	29	89
Both	b5	Get Them Tested	13	97	19	94	32	95
Both	b6	Kent C Card	10	92	0	-	-	-
Both	b7	KIS-SK	6	83	0	-	-	-
Both	b8	KYSH - Know Your Sexual Health	9	96	12	100	21	98
Both	b9	My Sex Doctor	14	100	32	89	46	94
Both	b10	NeedTayKnow	14	100	12	100	26	100
Both	b11	SexPositive	9	96	17	86	26	91
Both	b12	Sexual Health Guide	17	98	23	91	40	94
Both	b13	Sexual Health Liverpool	10	100	3	83	13	92
Both	b14	Your rapid diagnosis STD	24	76	43	68	67	72
Both	b15	Your Choice Your Voice (YCYV)	10	100	44	72	54	86

Web Table 19: Additional Information (iOS)

Platform	App ID	Title of App	App allows interaction with a healthcare profession (HCP)	Type of HCP	Contact via email	Contact via phone	Contact via app	Able to upload photo	Able to share info on sexual partners	Any other comments
iOS	i1	Sexually transmitted disease (STD) triage	Yes	Doctor	Yes	No	Yes	Yes	No	Clinic locator not accurate or complete, only private clinics. Service provision = send picture of skin concern and get answer from dermatologist within 24h plus clinic locator
iOS	i2	STD Guide	No	-	-	-	-	-	No	
iOS	i3	STD Glossary	No	-	-	-	-	-	No	Under definition of HPV they have the definition of HIV
iOS	i4	iCondom Coventry	No	-	-	-	-	-	No	Information on where to access condoms but no information on how, why & when you need to use
iOS	i5	99 - The Talk	No	-	-	-	-	-	No	
iOS	i6	Safer Sex	No	-	-	-	-	-	No	Only basic facts on contraception: success rate, brief method of action, effective against STD/pregnancy. No side effects, how to use. States emergency contraceptive pill is two pills 12hours apart. Vaguely implies condoms are only method of preventing STIs
iOS	i7	STD411	Yes	Unknown	Yes	Yes	Yes	No	No	Simple information on STDs including symptoms. No diagnosis or management.
iOS	i8	Private Girl Tips	No	-	-	-	-	-	No	States to be yeast infection must have itching AND white discharge, wrongly encourages use of baby powder 'down there'. Monistat is the recommended treatment. States Pap smear to be done yearly (USA).
iOS	i9	SWISH APP	No	-	-	-	-	-	No	
iOS	i10	Sex Health Dictionary & Sexual Health Video Lessons	No	-	-	-	-	-	No	
iOS	i11	Safesex Guide	Yes	Unknown	Yes	No	Yes	No	No	NSU described as 'urethral catarrh'
iOS	i12	Safe sex	No	-	-	-	-	-	No	
iOS	i13	SafeSex101	No	-	-	-	-	-	No	Although there isnt a facility to share with partners, the app does have a forum to speak to others about "topics" under a lesson section. Lessons & information section doen't work
iOS	i14	SAFE - Safety Awareness for Everyone	No	-	-	-	-	-	No	
iOS	i15	iSex - Sex Education and Terminology	No	-	-	-	-	-	No	In definitions it signposts you to terms that do not exist in the dictionary (e.g. genital herpes) or to a term that you are already in (e.g. diaphragm). In gonorrhoea it tells you about rare complications (without mentioning that they are rare) but doesn't mention common symptoms or the fact that it can be asymptomatic
iOS	i16	Girls's guide for sex myths	Yes	Unknown	Yes	No	No	No	No	
iOS	i17	CaSH 2 U	Yes	Unknown	No	Yes	No	No	No	Linked to SeX Factor ICE
iOS	i18	Pap Test Lite	No	-	-	-	-	-	No	
iOS	i19	Natural Yeast Infection Solutions	No	-	-	-	-	-	No	Very wordy and opinionated. Implies that medical practitioners are likely to mismanage the condition and treat with antibiotics. Potentially dangerous in terms of treatment options.
iOS	i20	A woman's guide to yeast infections	No	-	-	-	-	-	No	Very similar to Natural Yeast Infection Solutions by Minervaz

**HCP, health care professional; SP, sexual partner; ** Monistat a brand name for miconazole vaginal cream*

Web Table 20: Additional Information (Android)

Platform	App ID	Title of App	App allows interaction with a healthcare profession (HCP)	Type of HCP	Contact via email	Contact via phone	Contact via app	Able to upload photo	Able to share info on sexual partners	Any other comments
Android	a1	Abnormal Vaginal Discharge	No	-	-	-	-	-	Yes	Able to click to send email containing the information to someone else. Focus on fertility treatments as well as abnormal discharge which seems strange as the two are not necessarily related.
Android	a2	About Herpes Simplex Infection	No	-	-	-	-	-	No	Quite a lot of scare mongering. Makes herpes sound really horrendous.
Android	a3	After Sex	No	-	-	-	-	-	No	Very good. Links to a website for more information.
Android	a4	Bacterial Vaginosis Disease	No	-	-	-	-	-	No	Pictures/photos with text are incongruous - e.g. on page titled 'definition' there is a picture of a CT scanner
Android	a5	Bacterial Vaginosis Guide	No	-	-	-	-	-	No	Quite scaremongering. Lots of suggestions for natural remedies and natural prevention methods.
Android	a6	Bacterial Vaginosis Treatments	No	-	-	-	-	-	No	Links to external websites included in the app and to You Tube videos.
Android	a7	Chlamydia Disease and Symptoms	No	-	-	-	-	-	No	
Android	a8	Chlamydia Know it Prevent it Treat it	No	-	-	-	-	-	No	Links to website. No content within actual app.
Android	a9	Deadly Herpes Virus Acyclovir	No	-	-	-	-	-	No	
Android	a10	Female Herpes	No	-	-	-	-	-	No	Good information about herpes but then under treatment there are two links to websites selling remedies. Mainly discusses using culture for diagnosing HSV with only a brief mention of PCR
Android	a11	Genital herpes guide	No	-	-	-	-	-	No	Has game at end. One section links to wikipedia with no obvious relevance. Poorly written and contains very little information. Only mentions HSV-2 as a cause of genital herpes.
Android	a12	Genital Herpes Information	No	-	-	-	-	-	No	Excellent accurate comprehensive app which includes what to expect when you see a healthcare professional, coping support, pregnancy, & episodic & suppressive treatment.
Android	a13	Genital Herpes Treatment	No	-	-	-	-	-	No	Same info as genital herpes Ashi
Android	a14	Genital Herpes Treatment	No	-	-	-	-	-	No	Linked to genital herpes guide. Only mentions HSV-2 as a cause of genital herpes. Game at end
Android	a15	Genital Warts Guide	No	-	-	-	-	-	No	Scare-mongering e.g. 'Genital warts are bad. If they form in a bunch on your genitals, you will have a very bad time getting them treated and your relationships will shatter'. Same format as genital warts info.
Android	a16	Genital Warts Guide	No	-	-	-	-	-	No	Contains very little information. Same format and very similar to Havana app
Android	a17	Genital warts info	No	-	-	-	-	-	No	Virus never leaves the body. Much easier to prevent transmission of the virus than to deal with the virus after you have caught it. Multiple inaccuracies & scare-mongering. Link to more information (wikipedia) doesn't work. Game at end.

Web Table 20 continued

Platform	App ID	Title of App	App allows interaction with a healthcare profession (HCP)	Type of HCP	Contact via email	Contact via phone	Contact via app	Able to upload photo	Able to share info on sexual partners	Any other comments
Android	a18	Genital Warts Info	No	-	-	-	-	-	No	Exactly the same as Genital Warts Info by Havana apps
Android	a19	Genital Warts Info	No	-	-	-	-	-	No	Similar format to Genital warts info by Havana apps. Link to extra information takes you to a wikipedia
Android	a20	Genital Warts Information	No	-	-	-	-	-	No	
Android	a21	Get Rid of Bacterial Vaginosis	No	-	-	-	-	-	No	Scare mongering. Suggested management includes douching with grapefruit seed extract or hydrogen peroxide. Recommends accessing the bacterial vaginosis freedom guide for further info.
Android	a22	Get Rid of Yeast Infection Now !	No	-	-	-	-	-	No	Very similar to itune yeast apps
Android	a23	Gonorrhea Disease & Symptoms	No	-	-	-	-	-	No	
Android	a24	Guide to STDs	No	-	-	-	-	-	No	When something is sexually transmitted, it means that it has a very significant possibility of being transmitted being animals or human beings through sexual behaviour'. 'By sexual behaviour it does not only mean having vaginal intercourse. In fact, homosexuals can obtain this dreaded disease too through anal and oral sex'. 'Actually, it was only around the 1990's when scientists and medical professionals decided to call sexually transmitted diseases as the venereal diseases'. Refers to STDs as 'a disease' as opposed to individual infections that cause disease. 'Most of the strains of the human papillomavirus cause the onset of cervical cancer...' 'Once women are left untreated with Chlamydia, they become highly likely of acquiring HIV or the human immunodeficiency virus'. Re gonorrhoea: 'a huge percentage of the men who have acquired the infection do not exhibit symptoms.' 'Leaving gonorrhea untreated will only affect the infected person's joints and heart valves'. Re Trichomonas: 'The great thing about this disease is that it is the number one most curable sexually transmitted disease in the whole world' & 'Many believe that performing a natural douche once in a day while having a warm bath is very helpful. Actually, they are right. But this method will be much more effective is the juice of a single lemon is added as it increases the liquid's parasite-killing power'. 'Those who usually get infected with the genital herpes virus are the poor, those who are addicts with cocaine, those with multiple sexual mates, and also those who are uneducated'.
Android	a25	Herpes Knowledge	No	-	-	-	-	-	No	Very similar format to Ashi. Discusses genital herpes only being caused by HSV-2. Infers that you get it by having 'careless casual sex with strangers'. Information on how HSV is transmitted is ambiguous. Read more tab links to herpes simplex page on wikipedia. Game at end.
Android	a26	Herpes Lupus Psoriasis Eczema	No	-	-	-	-	-	No	Describes HSV-3 (zoster/shingles)

Web Table 20 continued

Platform	App ID	Title of App	App allows interaction with a healthcare profession (HCP)	Type of HCP	Contact via email	Contact via phone	Contact via app	Able to upload photo	Able to share info on sexual partners	Any other comments
Android	a27	Herpes Treatment	No	-	-	-	-	-	No	Both the prescription drug Valtrex and some medicinal herbs have been proven to reduce herpes viral shedding in clinical studies..' 'Certain medicinal herbs may also be beneficial in creating a strong immune resistance against HSV in non-infected partners'. Incubation period 2-12 days. Women with HSV-2 genital herpes the chance of spreading the virus to a man if they abstain from having sex during outbreaks is approx 3% in a year; for a man to woman - 8%. Video. Estimated 1:4 people in the UK are diagnosed with genital herpes. Genital herpes usually caused by HSV-2. Diet and certain foods can trigger outbreaks. Herpes virus does not pass through latex condoms. If you know that you have come into contact with the virus in the past few minutes or so then simple act of washing the infected area with soap and warm water can help to sweep away the virus from your hands and other areas. By doing this you can help avoid the virus from spreading further. Doesn't mention PCR testing (does mention viral culture, serologic tests and antigen tests). Discusses dietary habits and herbal remedies as ways of managing the infection. Although this app contains a lot of information that is accurate it also contains a lot of information that they claim is backed by evidence and it isn't
Android	a28	HPV Infection Information								
Android	a29	iGirl	No	-	-	-	-	-	No	Excellent comprehensive app. Crashed on 2 occasions. Centres based in Uganda.
Android	a30	Knowledge of Herpes	No	-	-	-	-	-	No	Exactly the same as Herpes Knowledge by Gooplay Apps. Very similar format to Ashi. Discusses genital herpes only being caused by HSV-2. Infers that you get it by having 'careless casual sex with strangers'. Information on how HSV is transmitted is ambiguous. Read more tab links to herpes simplex page on wikipedia. Game at end.
Android	a31	No Worries	Yes	Unknown	No	Yes	No	No	No	Comprehensive coverage of certain STIs and contraceptive methods including emergency contraception. Only app to mention both Ellaone and the IUD as methods of emergency contraception. Also discusses pros and cons of certain contraceptive methods.
Android	a32	NORISKS	Yes	Unknown	No	Yes	No	No	No	
Android	a33	Painful urination in men	No	-	-	-	-	-	No	Uses the term venereal diseases. Able to answer questions re symptoms but repeatedly got pop up box stating 'This application is not licensed. Please purchase it from Android Market' even though I'd paid £4.11 for the app.
Android	a34	Pelvic inflammatory disease	No	-	-	-	-	-	No	
Android	a35	Protection - Sex	No	-	-	-	-	-	No	Initial facts and figures quoted area out of date (references date from 2001) & they have NHS direct number at end rather than 111. In format of leaflet which is not particularly easy to scroll through
Android	a36	Pubic Lice Crabs Information	No	-	-	-	-	-	No	
Android	a37	SAFE	No	-	-	-	-	-	No	Exactly the same as iTunes SAFE app.

Platform	App ID	Title of App	App allows interaction with a healthcare profession (HCP)	Type of HCP	Contact via email	Contact via phone	Contact via app	Able to upload photo	Able to share info on sexual partners	Any other comments
Android	a38	Safer sex	No	-	-	-	-	-	No	Not obvious that you need to scroll through it. Clearly designed as leaflet/booklet and has just been transferred straight in to an app.
Android	a39	Samedaydoctor - STD Testing	Yes	Yes	Yes	Yes	Yes	No	No	
Android	a40	Sexual Education	No	-	-	-	-	-	No	Coitus interuptus - can work to prevent pregnancy if done right'
Android	a41	Sexually transmitted Stds	No	-	-	-	-	-	No	Cervical & STI screening is for USA not UK
Android	a42	Sheffield SH	No	-	-	-	-	-	No	Information on condoms links to a You Tube video. Able to send a text to order a chlamydia test kit via the app
Android	a43	STD glossary	No	-	-	-	-	-	No	Contains errors - e.g. definition of male condom = itch; Definition of CT discusses trachoma but fails to
Android	a44	Stop Vaginal Odor	No	-	-	-	-	-	No	The result of PID is a heavy discharge with an extremely noticeable bad smell'. Re Gonorrhea - 'this sexually transmitted disease causes a pus-like discharge that is accompanied with a rotten odor. It's possible to become re-infected even after each partners has been treated for it'. Re Chlamydia - it can also pass to an unborn baby during pregnancy'. Advises using tea tree oil, garlic & panty liners
Android	a45	Syphilis Disease and Symptoms	No	-	-	-	-	-	No	
Android	a46	The Big Book - Symptoms of STD	No	-	-	-	-	-	No	
Android	a47	The Sex Guide	No	-	-	-	-	-	No	Contact numbers for different services are out of date
Android	a48	Treat Genital Herpes Naturally	No	-	-	-	-	-	No	Offer of free video if you provide email address. Very small font.
Android	a49	Trichomoniasis information	Unknown	-	-	-	-	-	No	
Android	a50	UCT Safe Sex	No	-	-	-	-	-	No	
Android	a51	Yeast Infection	No	-	-	-	-	-	No	Candida (found in yeast infections) can infect your blood, causing an overload of toxins to disrupt your system, wreaking havoc on your mind and body. App finishes by saying that there is a really helpful guide/report but not stating which one this is and not discussing how vulvovaginal candiasis can be treated.
Android	a52	Yeast Infection Home Remedy	No	-	-	-	-	-	No	The symptoms they describe could also be HSV - e.g. 'burning and tingling sensation' 'make walking, switching positions, urinating and sexual intercourse difficult'. 'Sufferers are more likely to develop allergies inhaling airborne mold. Damp, dark locations can make them feel worse. They may also display a craving for sugar, breads, carbohydrates & alcohol, though sufferers may not necessarily be tolerant to alcohol. But even then these symptoms altogether may make you a candidate for another infection called bacterial vaginosis, which merits its own article.' 'Also, when left unchecked, thrush may cause dangerous side-effects, such as endometriosis, ovarian dysfunction and the release of toxins which may further jeopardize your immune system'. 'But if the yeast in our bodies reach more than the normal levels, that's when yeast infection strikes. Yeast infection causes our bodies to produce too much yeast and is triggered by a fungus called Candida albicans'. 'Vaginal yeast infection happens more to women after menopause'. Treatment and prevention includes dietary advice, apple cider vinegar solution, garlic, cranberry pills, olive leaf and grapefruit seed extract mixed together, raw garlic juice, hydrogen peroxide. Article based on book 'yeast infection no more' by Linda Allen. Repetitive & very lengthy.

Web Table 21: Additional Information (Both platforms)

Platform	App ID	Title of App	App allows interaction with a healthcare profession (HCP)	Type of HCP	Contact via email	Contact via phone	Contact via app	Able to upload photo	Able to share info on sexual partners	Any other comments
Both	b1	C&SH Somerset	2	-	-	-	-	-	2	Partner notification and treatment is only mentioned with regards to pubic lice, scabies and gonorrhoea. Specific names of drug treatments not mentioned. Provides information on where to acquire condoms and GUM services, but does not emphasise importance of regular check up
Both	b2	Conifer Sex Health	1	Unknown	2	1	2	2	2	
Both	b3	FPA - Find a Clinic	2	-	-	-	-	-	2	Information an all STIs not actually in app but in downloadable leaflets. I have included this information as it was very easy to download the leaflets within the app.
Both	b4	FREE 2 B ME	1	Unknown	2	2	1	2	2	NHS branded app. Vaguely mentions condoms are only method of preventing STIs. STIs described generally specifically mentioned in examples only. Diagnostic tests not expanded further than swabs and urine tests. No management. 'Without treatment, some STIs such as Chlamydia, HIV, Herpes, HPV and Hepatitis may stop you having a baby naturally' 'Most STIs can be treated easily with medicine. But, you should always use a condom as some STIs such as HIV, Herpes, HPV and Hepatitis have no cure, and if the symptoms are left untreated could cause infertility"
Both	b5	Get Them Tested	1	Unknown	1	1	2	2	1	Unable to access partner notifciation module unless you have attended their clinic. Incorrectly states HPV is bloodborne
Both	b6	Kent C Card	2	-	-	-	-	-	2	Cites 'is your wee dark, or does it smell bad?' as a reason to get tested for STIs. Doesn't mention asymptomatic infection. Location of clinic services, condom dispensers. Contact information of services.
Both	b7	KIS-SK	2	-	-	-	-	-	2	Doesn't mention copper coil role in EC. Doesn't mention IUS. % quoted aren't accurate. Questions/answers on contraception & safe sex. Clinic finder for contraception & STIs. States OCP is taken everyday, no mention of 7day break. States patch worn for 7day, replaced weekly. Success rates of some contraceptive methods are much lower than those stated in NHS choices
Both	b8	KYSH - Know Your Sexual Health	2	-	-	-	-	-	2	Descriptions of STIs basic eg few example symptoms. Only mentions antivirals for herpes, no management for other STIs. No mention of avoiding STI, use of condom, safe sex. General info on tests - bloods, swabs, urine.

Web Table 21 continued

Platform	App ID	Title of App	App allows interaction with a healthcare profession (HCP)	Type of HCP	Contact via email	Contact via phone	Contact via app	Able to upload photo	Able to share info on sexual partners	Any other comments
Both	b9	My Sex Doctor	2	-	-	-	-	-	2	Drug management is not specifically named, simply as antibiotics. Description of NGU: 'Acronym for nongonococcal urethritis, an infection caused by bacteria. The areas primarily affected by NGU are the cervix and the urethra. Typical symptoms are discharge from the penis or vagina and a burning sensation during urination. The infection can be sexually transmitted'
Both	b10	NeedTayKnow	2	-	-	-	-	-	2	Specific treatment names not mentioned; only antibiotcs, antivirals, cream. Does not mention cervical cancer risk of HPV. No mention of how often to get tested. Diagnostic tests not explained further than generalised 'pee in a pot', swabs and blood.
Both	b11	SexPositive	2	-	-	-	-	-	2	Link from herpes page doesn't work. Information on chlamydia inaccurate and scaremongering. Risk tool not accurate. STD description included transmission, symptoms and complications. Nothing on diagnosis or management. Doesn't specify how often you should get STD check.
Both	b12	Sexual Health Guide	2	-	-	-	-	-	2	Diagnostic tests mentioned as swabs, bloods or urine sample. Treatments mentioned only as antibiotics, antivirals, creams etc. Sources listed at end. Extensive list of contraceptives, dis/advantages, contraindications, success rates, MoA
Both	b13	Sexual Health Liverpool	2	-	-	-	-	-	2	Brief sentence each for different types of contraception. Very extensive list of available sexual health services. Does not mention what happens in STI testing, diagnostic tests or management of STIs. Briefly mentions chlamydia.
Both	b14	Your rapid diagnosis STD	4	-	-	-	-	-	2	No specific information on CT despite being the commonest STI. Drug managements are not specific to UK; dosage, routes, first-line etc incorrect. Incorrectly states warts are caused by HPV 1, Gardasil vaccine protects against HPV1 (not 11) and is approved for use in girls 9-26. Vaccine 100% effecitve in preventing infection with HPV types 16,18, 1 & 6. Herpetic urethritis occurs in 30-40% of affected men. Re candida vulvovaginitis: 'although transmission is thought to be mainly sexual, non-sexual infection can also occur'. Yeast may be visualised directly under the microscope using a KOH preparation'; 'ensure partner is treated to prevent reinfection'. Re NSU: 'Treatment should cover NG as this organism is present in about 50% of cases. ELISA test for CT. When doing the risk assessment, chlamydia doesn't come up as an option.
Both	b15	Your Choice Your Voice (YCYV)	4	-	-	-	-	-	2	The app links to a web application that provides people with more information on STIs and contraception. Able to join C card and order a chlamydia test online. BV, thrush and NSU are all discussed under the same section together e.g. 'If left untreated they can cause reduced fertility, inflammation of the joints, urethra and eyes, long-term pelvic pain, ectopic (outside the womb) pregnancy, blocked fallopian tubes, testicle and prostate infection'. Re HSV: 'This is called asymptomatic shedding or viral shedding and is extremely contagious'
